# The national atlas of tsetse flies and African animal trypanosomosis in Ethiopia

**DOI:** 10.1186/s13071-022-05617-9

**Published:** 2022-12-28

**Authors:** Tsegaye Gebre, Berisha Kapitano, Dagnachew Beyene, Dereje Alemu, Ahimedin Beshir, Zelalem Worku, Teshome Kifle, Ayana Selamu, Endalew Debas, Aschenaki Kalsa, Netsanet Asfaw, Weining Zhao, Massimo Paone, Giuliano Cecchi

**Affiliations:** 1 Animal Health Institute, Sebeta, Ethiopia; 2 Food and Agriculture Organization of the United Nations, Ethiopia Country Office, Addis Ababa, Ethiopia; 3 Livestock Development Institute, Addis Ababa, Ethiopia; 4 Animal Health Institute, Bedelle Animal Health Centre, Bedelle, Ethiopia; 5 Animal Health Institute, Asossa Animal Health Centre, Asossa, Ethiopia; 6 Animal Health Institute, Finote Selam Animal Health Centre, Finote Selam, Ethiopia; 7 Animal Health Institute, Arba Minch Animal Health Centre, Arba Minch, Ethiopia; 8 Food and Agriculture Organization of the United Nations, Animal Production and Health Division, Rome, Italy

**Keywords:** African animal trypanosomosis, Tsetse, Atlas, Epidemiology, Ethiopia.

## Abstract

**Background:**

With the largest cattle population in Africa and vast swathes of fertile lands infested by tsetse flies, trypanosomosis is a major challenge for Ethiopian farmers. Managing the problem strategically and rationally requires comprehensive and detailed information on disease and vector distribution at the national level. To this end, the National Institute for Control and Eradication of Tsetse and Trypanosomosis (NICETT) developed a national atlas of tsetse and African animal trypanosomosis (AAT) for Ethiopia.

**Methods:**

This first edition of the atlas focused on the tsetse-infested areas in western Ethiopia. Data were collected between 2010 and 2019 in the framework of national surveillance and control activities. Over 88,000 animals, mostly cattle, were tested with the buffy-coat technique (BCT). Odour-enhanced traps were deployed in approximately 14,500 locations for the entomological surveys. Animal- and trap-level data were geo-referenced, harmonized and centralized in a single database.

**Results:**

AAT occurrence was confirmed in 86% of the districts surveyed (107/124). An overall prevalence of 4.8% was detected by BCT in cattle. The mean packed cell volume (PCV) of positive animals was 22.4, compared to 26.1 of the negative. *Trypanosoma congolense* was responsible for 61.9% of infections, *T. vivax* for 35.9% and *T. brucei* for 1.7%. Four tsetse species were found to have a wide geographic distribution. The highest apparent density (AD) was reported for *Glossina pallidipes* in the Southern Nations, Nationalities and People's Region (SNNPR) (3.57 flies/trap/day). *Glossina tachinoides* was the most abundant in Amhara (AD 2.39), Benishangul-Gumuz (2.38), Gambela (1.16) and Oromia (0.94) regions. *Glossina fuscipes fuscipes* and *G. morsitans submorsitans* were detected at lower densities (0.19 and 0.42 respectively). Only one specimen of *G. longipennis* was captured.

**Conclusions:**

The atlas establishes a reference for the distribution of tsetse and AAT in Ethiopia. It also provides crucial evidence to plan surveillance and monitor control activities at the national level. Future work on the atlas will focus on the inclusion of data collected by other stakeholders, the broadening of the coverage to tsetse-free areas and continuous updates. The extension of the atlas to data on control activities is also envisaged.

**Graphical Abstract:**

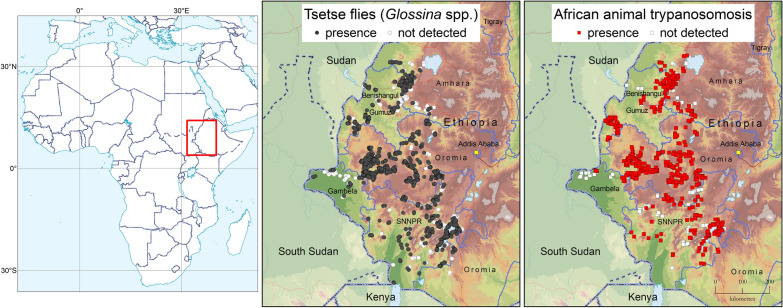

**Supplementary Information:**

The online version contains supplementary material available at 10.1186/s13071-022-05617-9.

## Background

Ethiopia has the largest livestock population in Africa, with an estimated 70.3 million cattle, 42.9 million sheep, 52.5 million goats, 11.3 million equines (2.1 horses, 8.9 donkeys and 0.3 mules) and 7.3 million camels [1]. Livestock resources contribute 45% of the agricultural gross domestic product (GDP) and 19% of the total GDP [2]. Further development of the livestock sector is one of the key targets of the Growth and Transformation Plan (GTP), the development strategy aiming to project Ethiopia to middle-income country status by 2025. However, the livestock sector falls far short of achieving its full potential. High disease prevalence, inadequate feed supply, poor genetic resources and poor marketing are the main bottlenecks for the development of the livestock sector in Ethiopia [3].

African animal trypanosomosis (AAT), also known as ‘nagana’, is one of the major livestock diseases constraining agricultural production in sub-Saharan Africa [4, 5]. AAT is caused by unicellular parasitic protozoa called trypanosomes, which are transmitted by the bite of hematophagous tsetse flies (Genus: *Glossina*). Among the many existing species of trypanosomes, *Trypanosoma vivax*, *T. congolense* and *T. brucei* have a particular economic relevance in livestock, and in particular in cattle [6]. Tsetse flies also transmit human African trypanosomosis (HAT), which is caused by two subspecies of *T. brucei* (i.e. *T. brucei gambiense* and *T. brucei rhodesiense*) [7]. In addition to tsetse flies, which are the sole cyclical or biological vectors of trypanosomosis, AAT can also be mechanically transmitted by other blood-sucking arthropods such as Tabanids and *Stomoxys* [8]. Notably, the mechanical mode of transmission enabled *T. vivax* to spread beyond sub-Saharan Africa and to become endemic also in Latin America [9].

Ethiopia is geographically located at the northeastern limit of the African tsetse belt [10]. Bioclimatic factors restrict tsetse distribution to the western part of the country, with a maximum longitude of 38 degrees East and a maximum latitude of 12° North [11, 12]. The lowlands bordering Sudan and South Sudan provide tsetse with favourable environmental conditions, but the flies have also progressively spread into the long, often steep river valleys carved into the massifs and plateaux of central Ethiopia [13, 14]. Further eastward spread is constrained by the low temperatures characterizing the Ethiopian highlands, but also by the semidesertic climate of the eastern lowlands. In the 1970s, the altitude of 1600 m was assumed to be the breeding limit for tsetse in Ethiopia [11], but in the following decades flies have also been captured at altitudes that are close to 2000 m [14]. Based on the shifting altitude limits, and depending on whether breeding areas only or areas of dispersal are considered, the area of tsetse infestation in Ethiopia has been variously estimated over the years between 66,000 km^2^ and 220,000 km^2^ [13]. However, in the absence of recent and comprehensive data on tsetse occurrence, these estimates should be considered as ballpark figures.

At the time of developing the atlas described in this article, which includes data for the 10-year period 2010–2019, five regional states were affected by tsetse flies in Ethiopia. These were, from north to south and anti-clockwise, Amhara, Benishangul-Gumuz, Oromia, Gambela and the Southern Nations, Nationalities and People's Region (SNNPR). After 2019, additional regional states were established in Ethiopia, but in this paper all results and maps are based on the regional divisions that were in effect in the period of data collection between 2010 and 2019.

**Fig. 1:**
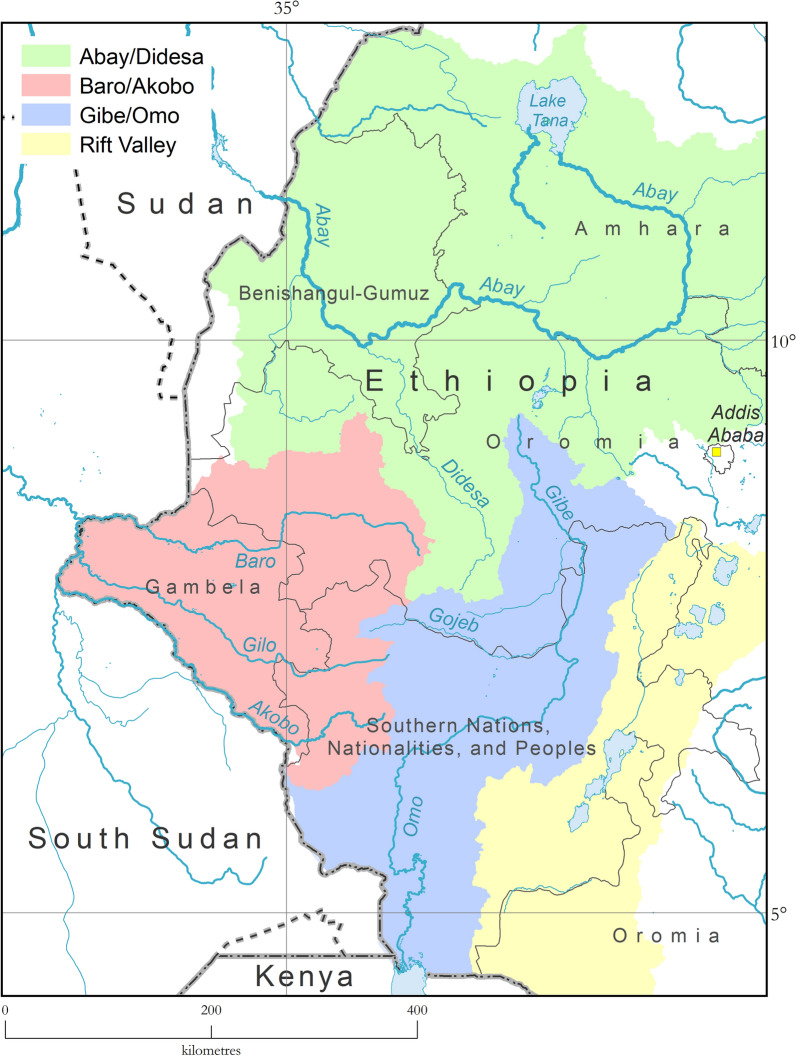
River basins influencing the geographic distribution of tsetse flies in western Ethiopia: Abay (Blue Nile)/Didesa, Baro/Akobo, Gibe/Omo and Rift Valley

Within the five regions mentioned above, four river basins or hydrological systems influence tsetse distribution in the country: the Abay (Blue Nile)/Didesa, Baro/Akobo, Gibe/Omo and Rift Valley (Fig. 1).

Six species of tsetse flies were historically recorded in Ethiopia: *Glossina pallidipes* and *G. morsitans submorsitans* of the savannah/morsitans group, *G. fuscipes fuscipes* and *G. tachinoides* of the riverine/palpalis group and *G. longipennis* and *G. brevipalpis* of the forest/fusca group [10, 15]. Of these, only the four species of the savannah and riverine groups have high economic importance and wide geographic distribution in the country [12, 13].

Compared to tsetse flies, AAT has a broader area of occurrence [13, 16]. This is especially the case for *T. vivax*, which is also reported from tsetse-free regional states such as Tigray and Afar [17–19], and it is believed to occur throughout the country [13]. In Ethiopia, the presence of *T. vivax* beyond the tsetse belt is mainly ascribed to mechanical transmission by non-tsetse vectors [20] and possibly also to the movement of animals between tsetse-free and tsetse-infested areas. Similar epizootic patterns are observed for *T. vivax* in several African countries [21, 22].

The severity of the AAT problem in Ethiopia is difficult to overstate. In particular, as of the 1970s, resettlement programmes moved large numbers of farmers into tsetse-infested areas, where they had to contend with the trypanosomosis challenge ever since [13]. The extremely high reliance of Ethiopian agriculture on draught-oxen also meant that, in the resettlement areas, trypanosomosis critically constrained crop production. Today, smallholders in tsetse-infested areas widely consider AAT as the major animal disease they have to grapple with. This has been well documented in SNNPR [23–26], western Oromia [25, 27, 28], western Amhara [29], Benishangul-Gumuz [30] and Gambela [25]. Beyond the tsetse belt, a severe AAT problem is also reported by farmers in Tigray and in the tsetse-free areas of Amhara, including an important problem of both curative and prophylactic drug misuse and drug resistance [18, 20, 31, 32]. Indeed, the use of drugs is often the only trypanosomosis control tool available to farmers [33], and drug usage can be extremely high. Six treatments per year are frequent [34], and up to 20 treatments in a year was reported for high-value oxen [35]. As opposed to AAT, HAT is not a major disease in Ethiopia [36–40].

**Fig. 2:**
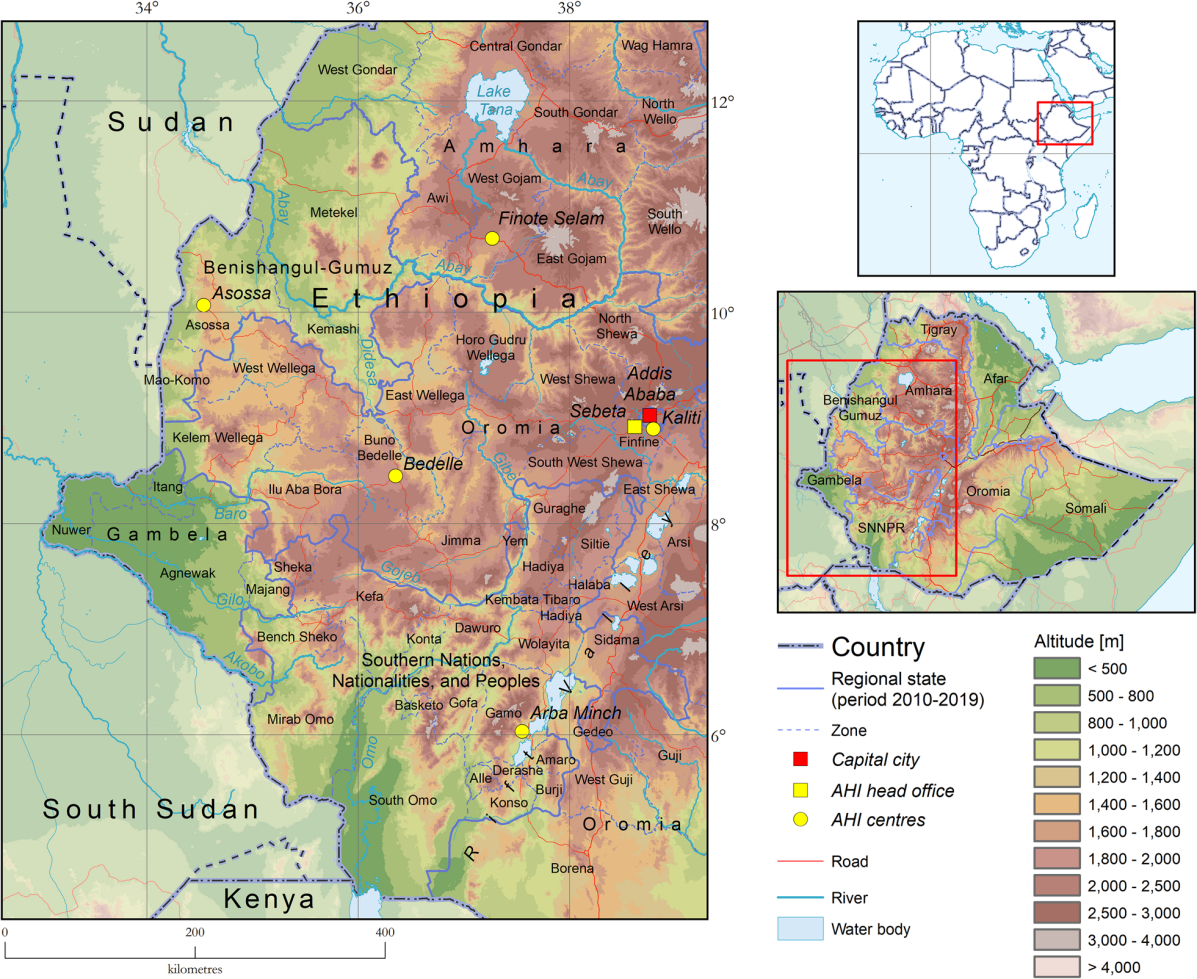
Animal Health Institute in Ethiopia, including the head office in Sebeta and its five Centres in Finote Selam (Amhara), Asossa (Benishangul-Gumuz), Kaliti (Addis Ababa), Bedelle (Oromia) and Arba Minch (SNNPR)

Because of the persisting challenge of tsetse and AAT, in 2013 the government of Ethiopia established the National Institute for Control and Eradication of Tsetse and Trypanosomosis (NICETT), a specialized national structure under the Ministry of Agriculture. NICETT’s mandate included the coordination of activities against tsetse and trypanosomosis at the national level, and its mission was to make affected areas free of the problem. Its ultimate goal was to increase the production and productivity of livestock and crops and to enhance food security and self-sufficiency of the affected populations. The institute was endowed with core staffing (approximately 300 members) and technical capacities. The overall government funding for NICETT was approximately 2.4 M USD per year (period 2018–2019), which was proof of Ethiopia’s commitment to the progressive control of trypanosomosis [41]. NICETT infrastructure included a central office and a tsetse mass-rearing facility in Kaliti (Addis Ababa) [42]. Furthermore, four regional offices were strategically placed in the tsetse-infested areas. These offices are located in Finote Selam (Amhara), Asossa (Benishangul-Gumuz), Bedelle (Oromia) and Arba Minch (SNNPR) (Fig. 2).

One of NICETT’s main responsibilities was to collect field data on the occurrence of tsetse and trypanosomosis. This information is crucial to target control activities at the national level and to assess their impact. Entomological and parasitological surveys were carried out both to generate a baseline before the start of control operations and for monitoring purposes during and after interventions. Over the years, these surveys have produced a vast amount of data. However, the lack of a centralized information system has severely hindered data analysis. To address this gap, NICETT developed a national atlas of tsetse and AAT. The initiative was technically supported by the Food and Agriculture Organization of the United Nations (FAO) through two projects [43, 44], which were implemented in the framework of the Programme Against African Trypanosomosis (PAAT) [45].

In 2022, NICETT was merged with the National Animal Health Diagnostic and Investigation Centre (NAHDIC) to create the Animal Health Institute (AHI). AHI was established with three goals: (1) animal health related research and diagnosis; (2) tsetse and trypanosomosis control and prevention; (3) animal health advisory and training service. The merger aimed to enhance the research, control, diagnostic and coordination capacity of NICETT, since NAHDIC was a long-standing animal health diagnostic and referral centre for Ethiopia and for the eastern Africa region. All mandates, roles and functions of NICETT were transferred to AHI (regulation number 503/2022). After the merger, NICETT central office was moved to Sebeta, and the four regional offices and the Kaliti mass-rearing facility were converted into AHI centres. Two additional AHI centres are planned to be established in Gambela and the recently established South West Ethiopia Peoples’ Region.

## Methods

The FAO continental atlas of tsetse and AAT provided the blueprint for the development of a national atlas in Ethiopia [12, 16]. National atlases previously developed in other countries (i.e. Sudan, Mali, Kenya, Zimbabwe and Burkina Faso) also provided additional methodological references [21, 46–49].

### Input data

The data on tsetse and AAT occurrence presented in this paper were collected by NICETT between 2010 and 2019 in the tsetse-infested areas of western Ethiopia. Data were collected either before the start of control activities (i.e. baseline data) or for monitoring purposes during or after interventions.

#### African animal trypanosomosis data

NICETT carried out trypanosomosis surveillance through its regional branch offices. The diagnostic method of choice was the buffy-coat technique (BCT) [50], and all AAT data presented in this paper are based on this parasitological test. A clinical diagnosis of AAT is often made by regional veterinary laboratories, district veterinary services and veterinary extension workers. However, these actors rarely use confirmatory tests and therefore they did not provide relevant information for the atlas. Universities, academic and research institutions also collect data on AAT in the context of research [16]. However, these data are not normally transmitted to the central veterinary authorities, and therefore they were not included in this first edition of the national atlas. Community-based animal health workers and livestock keepers make tentative diagnoses for the purpose of local treatment animals with trypanocides.

In Ethiopia, trypanosomosis surveillance normally focuses on bovines because of the heavy economic burden of the disease in these animals [51]. As a result, > 99.6% of the animals tested in the period 2010–2019 were cattle (the remaining 0.4% being sheep, goats and equines).

During surveys, the field teams usually perform site selection together with the district technical teams. Animals were randomly selected, and data on individual animals were recorded in hard copy datasheets. These include the owner’s name, administrative units [i.e. regional state, zone, district (*woreda*) and peasant association (*kebele*)], position (GPS-measured altitude, latitude and longitude), date of the survey, animal species, breed (‘local’ or ‘cross’), colour, sex, age, body condition (‘good’, ‘medium’ or ‘poor), packed cell volume (PCV) and the detected trypanosome species (i.e. *T. vivax*, *T. congolense*, *T. brucei* and mixed-infections thereof). The hard copy datasheets generated by field teams were subsequently entered into digital spreadsheets. These were first assembled by regional offices and then transmitted to the central office on a monthly, quarterly or annual basis.

#### Tsetse fly data

Tsetse surveys were carried out by NICETT’s regional offices with technical support from animal health extension workers at the district and peasant association level. Regional laboratories occasionally participate in the entomological surveys, especially in the event of AAT outbreaks. Local communities are also engaged, and they support field staff by transporting materials, opening access roads, clearing trapping sites and looking after the traps and targets.

Several types of traps were used in the study period, including NGU/NG2G [52], monopyramidal [53], bipyramidal [54], monoconical [55] and biconical [56]; sticky panels were also used [57]. Fly attractants like acetone, octenol, phenol, 3-week-old cattle urine or a combination thereof are used to enhance attractiveness for tsetse. Traps were deployed in suitable habitats for the flies, and their individual location was geo-referenced with GPS. Grease was smeared at the bottom of the trap poles to prevent ants hunting the captured flies, and vegetation was cleared for visibility in a radius of 2 m around the trap. As a rule, traps were maintained in position for 2 or 3 days (i.e. 48 or 72 h) before being removed. Flies were then collected from the cages and counted. Their species and sex were identified and recorded.

Field teams registered data from individual traps using standard recording sheets [58]. Recorded information includes the administrative units, GPS-measured geographic coordinates, trap type, vegetation type, odour attractant, start and end date of trapping, number of tsetse flies captured (disaggregated by species and sex) and number of other biting flies captured.

### The atlas development process

Capacity development provided by FAO was a key first step for the development of the atlas. The training focused on data collation, cleaning, harmonization, geo-referencing and handling. Another important enabling factor was the standardization of the field data recording formats, which were initially different across the centres. In terms of data flow, the field teams routinely capture data on paper recording sheets, which are subsequently entered into digital spreadsheets. The digital files are then transferred to the national office via email on a monthly, quarterly, semestral or yearly basis.

For the development of the atlas, special efforts were made systematically to collate existing data from decentralized centres and the head office. A digital repository was developed to centralize all input data. The repository is first organized by type of data (tsetse or AAT) and then by region, year, district and month of collection for easy data tracking, extraction and handling.

Following the data assembling in the repository, major efforts were devoted to data cleaning, harmonization and geo-referencing involving the head office, the centres and supported by FAO. Dates recorded in the Ethiopian calendar were converted to the Gregorian calendar. GPS-based geographic coordinates were harmonized to the GIS-ready standard ‘decimal degrees’. Potential data entry errors in the coordinates (i.e. outliers) were systematically checked in GIS and corrected if needed. Data were also checked for possible duplications.

### The database

The atlas database is divided into two components: one for entomological data and one for AAT data. Each component is stored in a separate Microsoft Excel file. In both files, each record is linked to the respective source file in the data repository.

#### Tsetse database

For the tsetse database, each row represents a single trap. The related information includes the region, zone, district (woreda), peasant association (kebele), village (locality), trap identifier (trap ID), trap type, altitude, latitude, longitude, vegetation type, odour attractant, start and end date of trapping, duration of trapping, number of tsetse flies captured, tsetse species, tsetse sex, number of other biting flies captured (tabanids and Stomoxys) and the source data file. When a single trap captured more than one tsetse species, separate records are entered for each tsetse species. The abundance of flies, or AD, is also recorded, and it is expressed as number of flies/trap/day.

#### AAT database

For the AAT database, each row represents a single tested animal. The related information includes region, zone, district (woreda), peasant association (kebele), village (locality), altitude, latitude, longitude, date of the survey, animal identifier (ID), animal species, breed, colour, sex, age, body condition, PCV, the detected trypanosome species, the diagnostic method, sampling method and the source data file name and the path thereof for easy tracking.

## Results

**Fig. 3:**
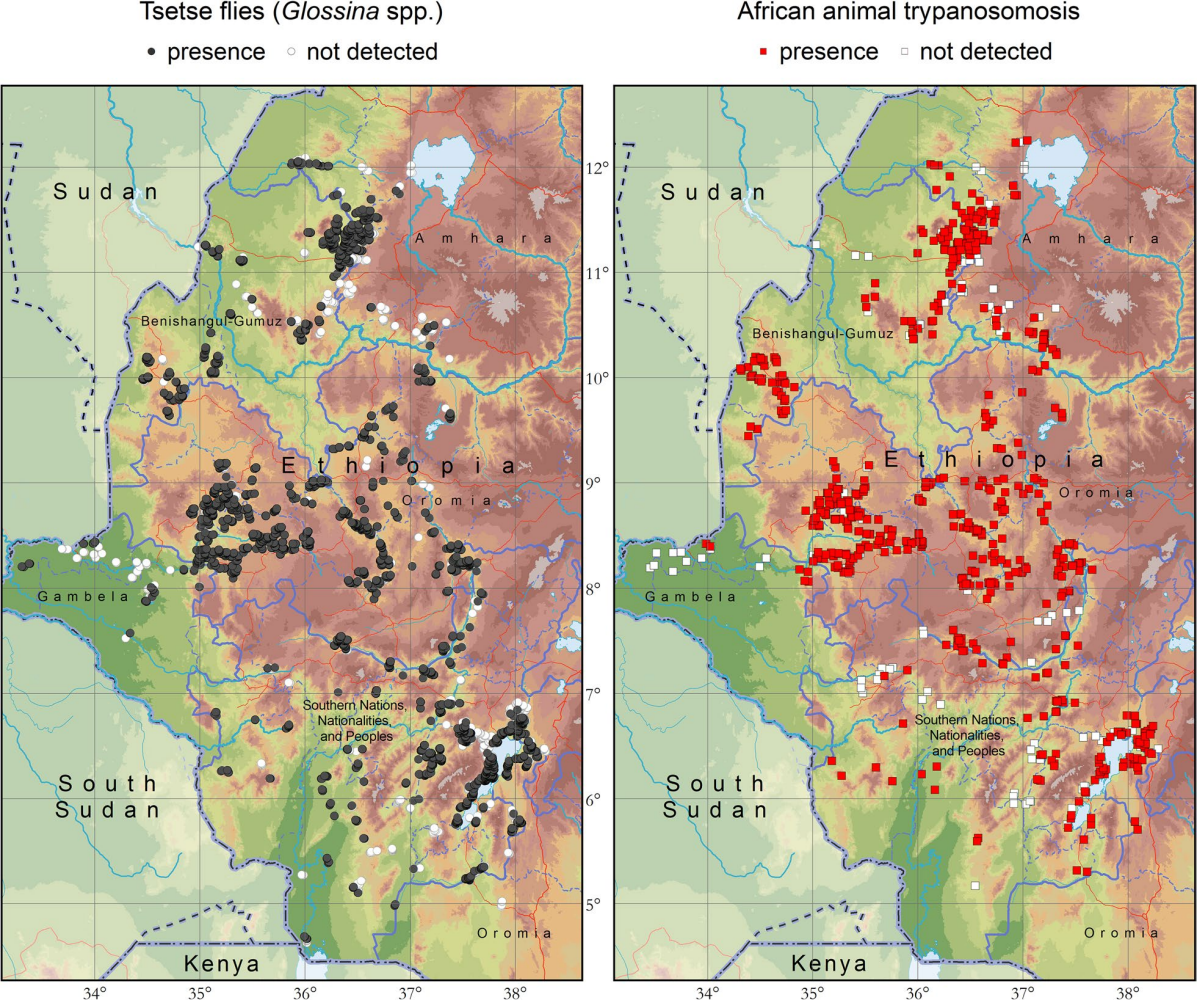
Reported geographic distribution of tsetse flies (genus *Glossina*) and African animal trypanosomosis in Ethiopia. Data collection period: 2010–2019

Figure 3 summarizes the atlas by showing the reported occurrence of tsetse flies and AAT for the period 2010–2019. Surveyed locations where tsetse and AAT were not detected are also included in the figure.

Figure 3 shows that the atlas provides a good coverage of the tsetse-infested areas in Ethiopia. Also, as field surveys normally address both tsetse and AAT at the same time, there is a close correspondence between the data coverage of the two components.

### Tsetse flies

**Fig. 4:**
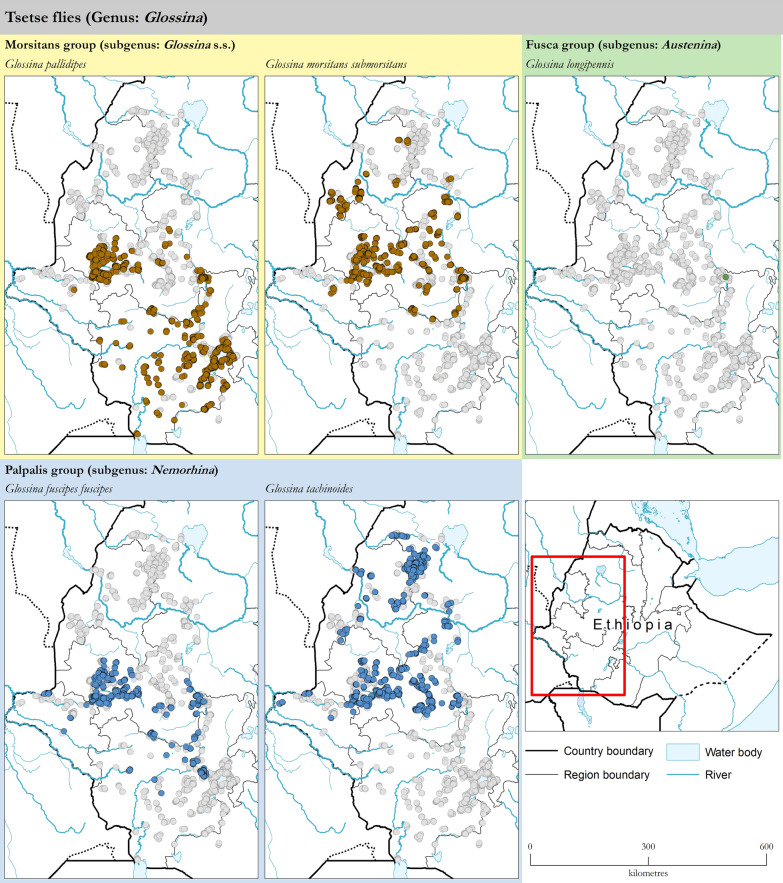
Presence (coloured circles) and absence (surveyed but not detected, grey circles) of tsetse fly species in Ethiopia. Data collection period: 2010–2019

A total of 16,865 trapping events were assembled in the atlas. These trapping events originated from 14,498 different locations, for a total intensity of 45,820 trap days. The AD, as measured by the average number of flies captured per trap per day, was 3.1 flies/trap/day. The results of these surveys, disaggregated by region, are summarized in Table 1, while results disaggregated by zone and by district are in Additional file 1: S1 and Additional file 2: S2, respectively. The geographic distribution of the different tsetse species in western Ethiopia is shown in Fig. 4, while higher resolution maps are available in Additional file 3: S3.

**Table 1:** Apparent density of tsetse flies in Ethiopia. Data collection period: 2010–2019

Region	Trapping locations [*n*]	Trapping events [*n*]	Trapping intensity [trap days]	Tsetse flies [flies/trap/day]					
				*G. pallidipes*	*G. morsitans submorsitans*	*G. fuscipes fuscipes*	*G. tachinoides*	*G. longipennis*	Genus: *Glossina*
Amhara	1377	1388	2818	0	0.02	0	2.39	0	2.42
Benishangul-Gumuz	2662	2771	5989	0	0.84	0	2.38	0	3.22
Gambela	457	457	1077	0.01	0.02	0.28	1.16	0	1.47
Oromia	5879	6687	19,133	0.81	0.73	0.28	0.94	0	2.75
SNNPR*	4123	5562	16,803	3.57	0	0.17	0	5.95 × 10^–5^	3.75
TOTAL	14,498	16,865	45,820	1.65	0.42	0.19	0.88	2.18 × 10^–5^	3.13

^*^Southern Nations, Nationalities and People's Region

The envelope of tsetse distribution in Ethiopia is confirmed to extend from the westernmost part of the country in Gambela to 38.2° East and from the southernmost tip of SNNPR to 12.0° North. Tsetse are also shown to penetrate deeply into the valleys of the Gibe/Omo, Baro/Akobo and Abay/Didesa River systems.

Four species of tsetse flies are confirmed to have a broad geographic distribution in Ethiopia, i.e. *G. pallidipes*, *G. morsitans submorsitans*, *G. fuscipes fuscipes* and *G. tachinoides*. In some areas in the Oromia region, all four species were found in the same locations. *Glossina longipennis* was also captured, albeit only as a single specimen in the Gibe River basin. *Glossina brevipalpis* was not detected in this study period.

*Glossina pallidipes* was found to have the highest AD. In particular, in the SNNPR, *G. pallidipes* was detected at an average apparent density of 3.57 flies/trap/day. In addition to being broadly distributed across the SNNPR, *G. pallidipes* was also abundant in western Oromia (AD 0.81) and detected in small areas in Gambela. The species was not detected in Benishangul-Gumuz and Amhara. Looking at the river basins and hydrological systems, *G. pallidipes* was found in the Rift Valley, Gibe/Omo and Baro/Akobo as well as in the upper reaches of the Didesa River. For *G. morsitans submorsitans*, the other species of the savannah group present in Ethiopia, the broadest distribution and highest densities were detected in Benishangul-Gumuz (AD 0.84) and western Oromia (AD 0.73). The species was also present at lower densities in Gambela (AD 0.02) and Amhara (AD 0.02), while it was not detected in SNNPR. In terms of river basins, *G. morsitans submorsitans* was found in the Baro/Akobo, Abay/Didesa and upstream parts of the Gibe/Omo.

As to the tsetse species of the riverine group, *G. fuscipes fuscipes* was detected in western Oromia (AD 0.28), Gambela (AD 0.28) and SNNPR (AD 0.17). Its distribution is centred around the Baro/Akobo basin, but it extends further east and south into the Didesa and Gibe-Omo. For *G. tachinoides*, apparent densities were at their highest in Amhara (AD 2.39) and Benishangul-Gumuz (AD 2.38), but the species was also widely distributed in western Oromia (AD 0.94) and present in Gambela (AD 1.16).

### African animal trypanosomosis

**Fig. 5:**
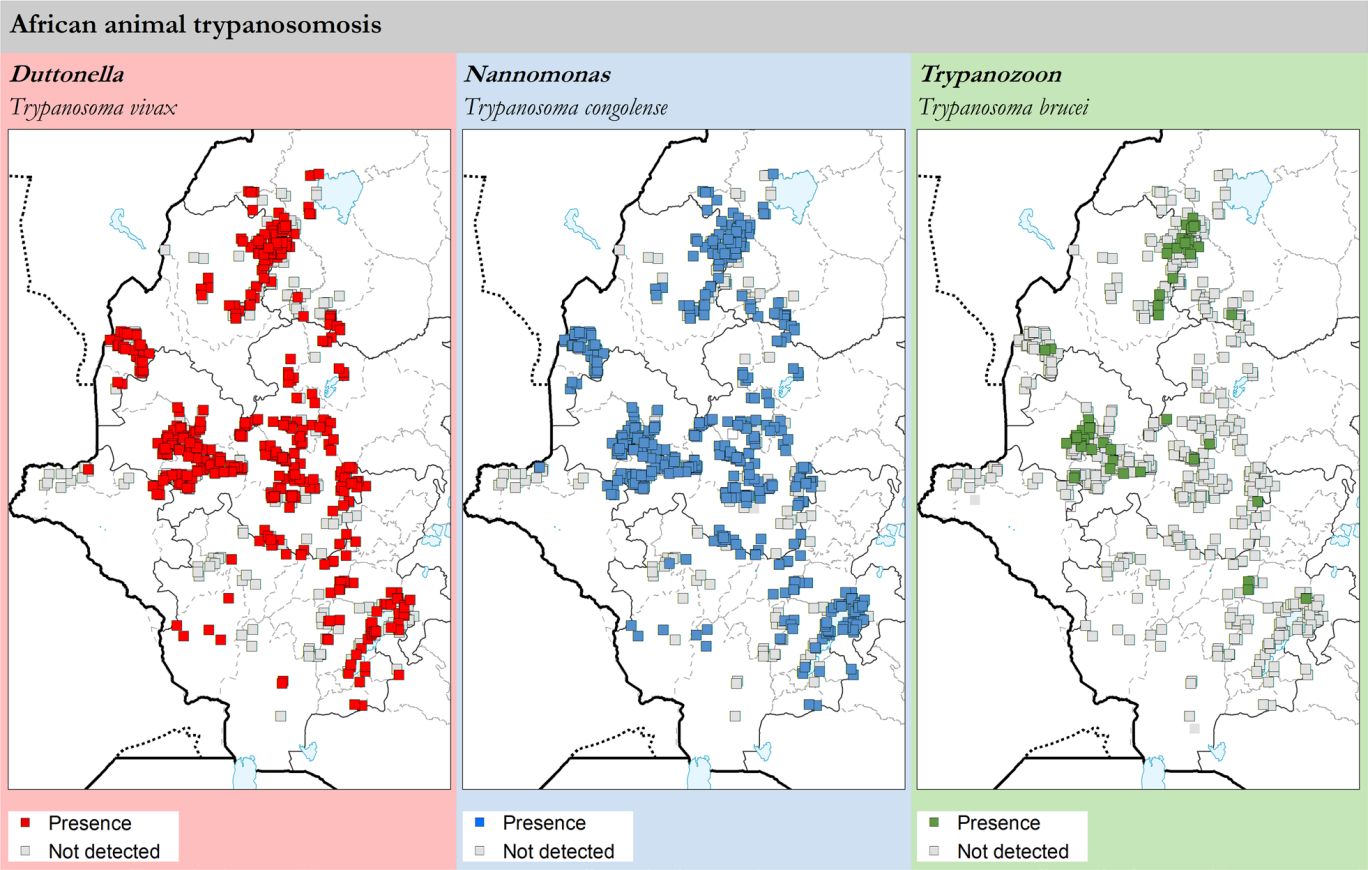
Presence (coloured squares) and absence (surveyed but not detected, grey squares) of *T. vivax*, *T. congolense* and *T. brucei* in cattle as determined with the buffy-coat technique (BCT). Data collection period: 2010–2019

For the period 2010–2019, data on the testing of 88,331 animals were assembled in the national atlas. Since 99.6% of these were bovines (88,003), from here on results are presented for cattle only. The results disaggregated by region are summarized in Table 2, while the geographic distribution of the different trypanosome species is shown in Fig. 5. Results disaggregated by zone and by district are in Additional file 4: S4 and Additional file 5: S5, respectively.

**Table 2:** Prevalence of bovine trypanosomosis in Ethiopia as determined with the buffy-coat technique (BCT). Data collection period: 2010–2019

Region	Animals tested	*T. vivax*		*T. congolense*		*T. brucei*		Total		Packed cell volume		
	[*n*]	[*n*]	[%]	[*n*]	[%]	[*n*]	[%]	[*n*]	[%]	[%]		
										Positive	Negative	All
Amhara	13,482	177	1.31	289	2.14	14	0.10	474	3.52	22.6	26.5	26.4
Benishangul-Gumuz	19,890	269	1.35	815	4.10	18	0.09	1072	5.39	22.9	26.3	26.1
Gambela	1824	5	0.27	9	0.49	0	0.00	14	0.77	23.2	29.8	29.8
Oromia	40,276	860	2.14	1153	2.86	36	0.09	2024	5.02	22.8	26.4	26.2
SNNPR*	12,531	248	1.98	419	3.34	6	0.05	657	5.24	20.4	24.0	23.8
TOTAL	88,003	1559	1.77	2685	3.05	74	0.08	4241	4.82	22.4	26.1	25.9

^*^Southern Nations, Nationalities and People's Region

A total of 4241 animals were found positive for trypanosomal infection, for an overall prevalence of 4.82%. These figures, and those provided in Table 2, Additional file 4: S4 and Additional file 5: S5, include mixed infections with more than one species of trypanosome. With the exception of Gambela, where testing was limited, parasite rates were remarkably similar in the different regions. AAT was confirmed in all but one of the 34 surveyed zones and in 86% of the surveyed districts (107/124). The impact of AAT on the haematocrit was sizable, with the mean PCV of affected animals being 22.4 compared to 26.1 of negative animals.

Close to two thirds of all infections were caused by *T. congolense* (2685, 61.9%) and more than a third by *T. vivax* (1559, 35.9%). Despite this difference, the two species seem to have a similar geographic distribution in western Ethiopia, both having been detected across the surveyed areas (Fig. 5). Indeed, both *T. vivax* and *T. congolense* were found in virtually all surveyed zones (32/34). The remaining small number of infections (74, 1.7%) were caused by *T. brucei*, which displayed a patchier pattern of detection (12/34 study zones).

### Database completeness

Entomological and epidemiological data without a geographical reference, and in particular without geographical coordinates, have not been included in the database yet, but kept separately for further checking and later incorporation. Therefore, data completeness was assessed only for fully georeferenced records. For both the tsetse and AAT databases, 100% completeness was achieved for administrative units (region, zone, district and peasant associations), while 46.39% and 9.2% village names were complete, respectively. For the tsetse database, tsetse species, trap ID, trap type, attractants used, survey period and trapping duration were 100% complete; high levels of completeness were also achieved for vegetation (91.21%), altitude (96.41%), tsetse intervention activity (71.64%) and data source files (95.31%). For the AAT database, the sampling method, data source file, date of survey, animal species and breed were 100% complete, whereas other elements like altitude, color, sex, age, body condition and PCV were 92.5%, 30.23%, 99.99%, 97.74%, 98.57% and 99.88% complete, respectively.

## Discussion

This first edition of the national atlas of Ethiopia provides a large scale and up-to-date reference on the distribution of tsetse flies and AAT across the tsetse-infested areas of the country. However, despite the vast amount of data collected and the large geographical coverage, the atlas is still affected by a number of gaps and limitations. We discuss the main ones in the following section. We then look at the findings in relation to the existing literature, with a focus on publications on tsetse and AAT occurrence in specific locations of Ethiopia [12, 16].

### Present limitations and gaps of the atlas

The lack of data on the occurrence of AAT in tsetse-free areas is arguably the main limitation of this first edition of the atlas. Tsetse-free areas in Ethiopia include the three northernmost and easternmost regions (i.e. Tigray, Afar, Somali, Harari regions and Dire Dawa city administration) as well as central, eastern and southern Oromia and large parts of Amhara. As documented in several countries, AAT and especially *T. vivax* can occur at large distances from the tsetse belt [21, 22]. *Trypanosoma vivax* is known to be widespread in Ethiopia [13, 59], and while the burden of AAT in tsetse-free areas is bound to be lower than within the tsetse belt, its impact should not be underestimated.

**Fig. 6:**
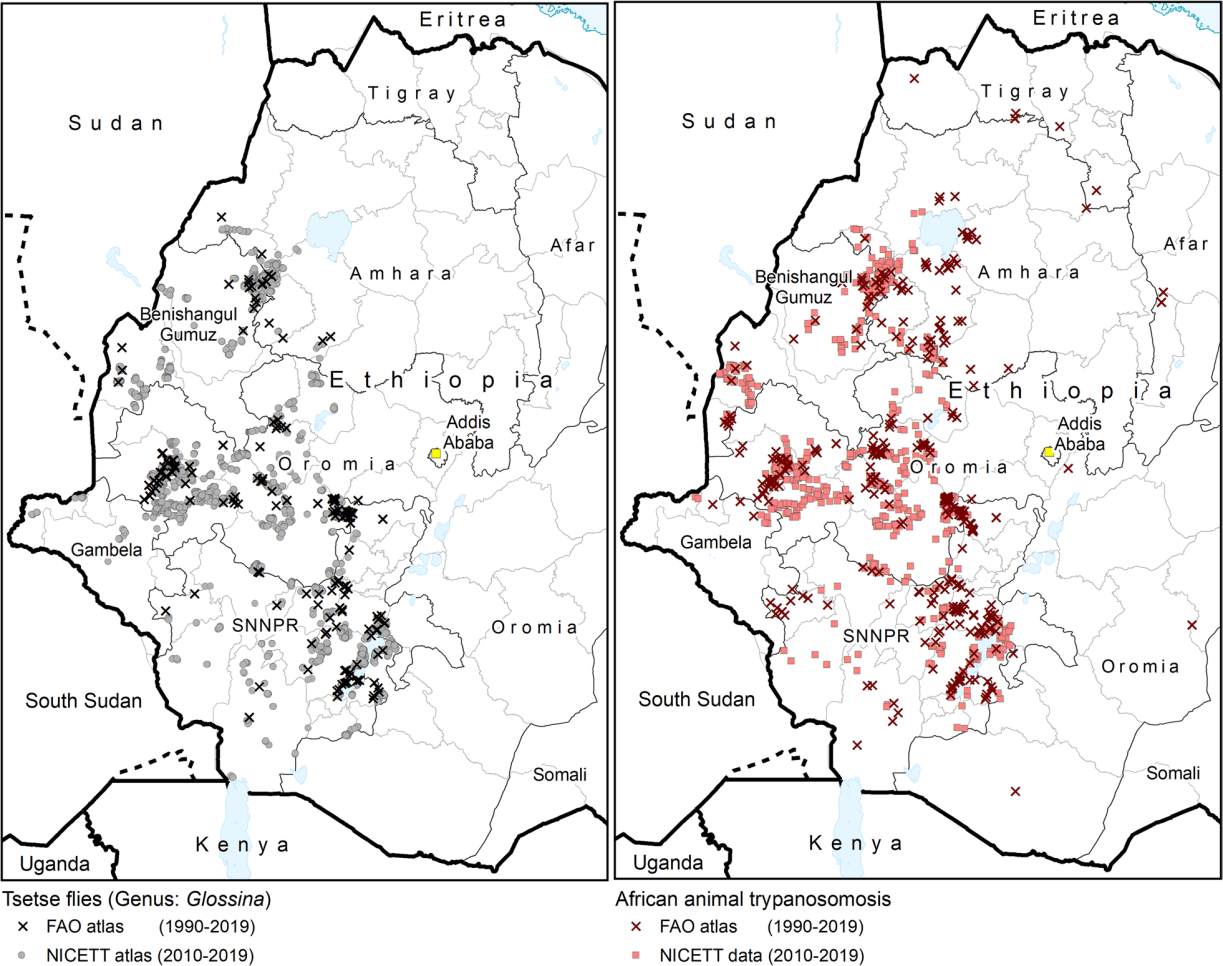
Data from the FAO continental atlas of tsetse and AAT (period 1990–2019) overlaid on the national atlas data (2010–2019). Data from the continental atlas are extracted through a review of scientific publications [12, 16]

Another major limitation of the atlas is that, to date, it does not incorporate data collected by universities and other academic or educational institutions. In the framework of the continental atlas of tsetse and AAT, FAO is in the process of mapping data from scientific publications for the whole of Africa [12, 16, 22], and Fig. 6 shows these data overlaid onto the national atlas in Ethiopia. All data extracted by FAO from publications are available for national authorities in Ethiopia to complement their national atlas; however, the raw data behind the papers are not available at FAO level. In particular, the information that can be extracted from the papers lacks the trap- and animal-level data upon which hinges the national atlas in Ethiopia. A proper inclusion of the published information into the national atlas will require the engagement of academic institutions, with a view towards including the raw data that underpin scientific publications.

The time coverage of 10 years is arguably another limitation of this first edition of the atlas. In fact, other existing national atlases cover periods of 15–20 years or more [21, 46–49]. A time frame longer than 10 years would allow temporal trends to be better captured [48], and it would also help fill some of the existing geographical gaps.

Looking at the AAT component of the atlas, one weakness is its total reliance on BCT as a diagnostic method. BCT is an effective technique when AAT diagnosis is geared towards control, as it is the case in the surveillance activities upon which the national atlas is based. In fact, BCT-positive animals are normally those showing more overt clinical symptoms and reduced PCV, and as such they represent good targets for treatment [60]. However, the use of more sensitive diagnostic tools such as serological or molecular methods would enhance knowledge of exposure to and diversity of pathogenic trypanosomes [61].

Another limitation of the national AAT database is the very limited data on host species other than cattle (< 0.5%). This is not a minor gap in a country with very high livestock numbers and diversity and where trypanosomosis is well known to affect also small ruminants [62], donkeys [63] and other livestock species [17].

Finally, the national atlas for Ethiopia lacks data on the occurrence of *T. evansi*, an important pathogenic, non-tsetse-transmitted trypanosome. *Trypanosoma evansi* causes the disease called ‘surra’ [64], which has a broad global distribution [65]. In Africa, surra mainly affects camels, and in Ethiopia the disease is present in the eastern, northern and southern parts of the country [17, 19, 66]. The inclusion of *T. evansi* infections in the national atlas would give a more complete picture of vector-borne animal trypanosomoses in the country.

### Occurrence of tsetse species

In this section we discuss our findings on the geographic distribution of tsetse species in Ethiopia in relation to the available literature.

*Glossina pallidipes* is the dominant tsetse species in many countries in eastern Africa [47, 67]. In western Ethiopia, our study found *G. pallidipes* to have the highest densities of all species and a broad distribution in two areas (i.e. SNNPR and western Oromia). The species was also captured in one area in Gambela, despite the relatively low sampling effort in this region, thus corroborating previous reports [68]. The absence of detection of *G. pallidipes* in Benishangul-Gumuz and Amhara is in line with historical and current knowledge [10, 12].

Regarding *G. morsitans submorsitans*, its continental distribution stretches from Senegal to Ethiopia. In many countries its populations have been shrinking because of land cover changes and the reduction of wildlife hosts [46, 69], and the species is now mainly confined to protected areas [22]. By contrast, our findings show that *G. morsitans submorsitans* maintains a fairly broad distribution in western Ethiopia, having been detected in all but one study regions (i.e. SNNPR). Furthermore, even in the SNNPR the species could still be present, having been reported from the Omo valley as recently as in 2015 [12, 70].

The populations of *G. fuscipes fuscipes* in Ethiopia are believed to be separated from the main belt in central Africa [10]. However, the species is reported across the border with Sudan [21], while recent information from South Sudan is very limited [71]. In our study the highest densities of *G. fuscipes fuscipes* were detected in Gambela, followed by Oromia and SNNPR. The absence of detection of this species in the Benishangul-Gumuz and Amhara regions, as well as in the Rift Valley basin, is consistent with current knowledge [12].

Even more markedly than for *G. fuscipes fuscipes*, the populations of *G. tachinoides* in Ethiopia are separated from the species’ main continental belt, which stretches from Guinea to the Central African Republic [10]. In our study, *G. tachinoides* was found to be the main species at the northern limit of the tsetse distribution in Ethiopia, and in particular in the Abay River basin in Amhara and Benishangul-Gumuz regions. The species is also widely distributed in Oromia and Gambela, while the absence of detection of *G. tachinoides* in SNNPR is consistent with current knowledge [12].

*Glossina longipennis* is known to be distributed in fairly fragmented populations in eastern Africa [10, 15], including recent reports from Kenya [12, 47] and Tanzania [67]. The species was also known to be present in Ethiopia [13], although it did not emerge in recent literature reviews [12]. In our study, the finding of one *G. longipennis* in the Gibe/Omo basin is consistent with past records [13], and it indicates that the species is likely to be persisting in Ethiopia at very low densities.

Finally, *G. brevipalpis* was known to be patchily distributed from South Africa to Ethiopia [10, 15], and recent reports confirm its presence from South Africa to Kenya [47, 67, 72, 73]. However, our study, consistently with recent literature reviews [12], did not find evidence of its persistence in Ethiopia.

### Occurrence of African animal trypanosomosis

The national atlas shows that AAT is widespread in the tsetse-infested regions of Ethiopia. The overall prevalence of 4.8% is in line with estimates from meta-analysis of published data [74], with the advantage of being based on a much larger and up-to-date dataset.

When compared with large-scale investigations of similar size, scope and diagnostic method, the observed prevalence for bovine trypanosomosis in western Ethiopia is shown to be higher than in tsetse-infested areas in Kenya (1.8% [47]), but lower than in Burkina Faso (6% [49]) and Mali (7% [46]). It is also lower than Nigeria, where a literature review yielded an 8.6% prevalence [22]. Regarding the relative proportion of *T. vivax* and *T. congolense*, the ratio between these two species that we found in Ethiopia (i.e. 0.58) was similar to that in Mali (i.e. 0.76) and Burkina Faso (i.e. 0.9), where *T. congolense* was also predominant. By contrast, *T. vivax* was relatively more prevalent in Kenya (ratio 1.14). As to *T. brucei*, its very low prevalence in Ethiopia is similar to the very low levels found in other countries [22, 46, 47, 49].

When interpreting the enzootic situation of AAT in western Ethiopia, it is important to note that it is influenced by past and ongoing control activities. A detailed discussion of past and present interventions carried out over the years is beyond the scope of this paper. However, notably, more than two-thirds of the tsetse-infested areas in Ethiopia are presently estimated to be at some level of control. Insecticide-treated cattle (ITC) is the most widely used control method [75], because of its cost-effectiveness in areas where a sufficient number of cattle are present [76]. Other tsetse-control methods deployed in Ethiopia include insecticide-treated targets [75], ground spraying, the sterile insect technique [42] and the sequential aerosol technique [77, 78]. Regarding the direct control of AAT through trypanocidal drugs, diminazene aceturate and isometamidium chloride are the most widely used compounds [34]. NICETT is active in the procurement, distribution and administration of trypanocides, but other stakeholders such as regional authorities and NGOs are also involved. Worryingly, aberrant use and poor quality of trypanocidal drugs are widespread in Ethiopia, and they pose a serious risk for drug resistance [34, 79, 80].

## Conclusions

AHI will continue to enhance and regularly update the national atlas of tsetse and AAT, and it will also make efforts to gather and include data collected by universities and other learning and research institutions. Future work should also tackle the issue of disease control data. Indeed, a large amount of information is available at AHI on the application of different tsetse and AAT control tools. However, as was the case for epizootic and entomological data before the atlas was developed, a system to centralize and map control data is lacking. When the latter is developed, it will be possible to combine epizootic, entomological and control data and thereby assess and monitor the progress in the control of AAT at the national level through the application of progressive control pathway (PCP) approach [41]. A number of these activities are being implemented in the framework of the recently launched project ‘COntrolling and progressively Minimizing the Burden of Animal Trypanosomosis’ (COMBAT) [81].

## Supplementary Information

**Additional file 1: S1.** Apparent density of tsetse flies in Ethiopia by zone. Data collection period: 2010–2019.

**Additional file 2: S2.** Apparent density of tsetse flies in Ethiopia by district (woreda). Data collection period: 2010–2019.

**Additional file 3: S3.** Presence and absence (surveyed but not detected) of tsetse fly species in Ethiopia. Data collection period: 2010–2019.

**Additional file 4: S4.** Prevalence of bovine trypanosomosis in Ethiopia by zone. Data collection period: 2010–2019.

**Additional file 5: S5.** Prevalence of bovine trypanosomosis in Ethiopia by district (woreda). Data collection period: 2010–2019.

## Data Availability

Relevant data are within the manuscript and its Supporting Information files. Point-level data, which are not within the manuscript and its Supporting Information files, are the property of the Government of Ethiopia. Requests for data can be addressed to: Tesfaye Rufael Chibssa, Director General, Animal Health Institute, PO Box: 04 Sebeta, Ethiopia, Phone: + 251 11 338 0898, Email: director@ahi.gov.et; info@ahi.gov.et. Requests will be evaluated on the basis of the national data policy.

## Abbreviations

AAT: African animal trypanosomosis
AHI: Animal Health Institute
BCT: Buffy-coat technique
FAO: Food and Agriculture Organization of the United Nations
GPS: Global Positioning System
HAT: Human African trypanosomosis
HQ: Head Quarters
ITC: Insecticide-treated cattle
ITT: Insecticide-treated targets
NAHDIC: National Animal Health Diagnostic and Investigation Centre
NICETT: National Institute for Control and Eradication of Tsetse and Trypanosomosis
PCP: Progressive control pathway
PCV: Packed cell volume
PAAT: Programme against African Trypanosomosis
SNNPR: Southern Nations, Nationalities and People's Region
WHO: World Health Organization
