# Supplementary material for: The national atlas of tsetse flies and African animal trypanosomosis in Ethiopia
Source: Parasit Vectors. 2022 Dec 28;15:491. doi: 10.1186/s13071-022-05617-9 (PMC9798648; doi:10.1186/s13071-022-05617-9)
Supplement: Supplementary file 1 — Additional file 1: S1. Apparent density of tsetse flies in Ethiopia by zone. Data collection period: 2010–2019. [file 13071_2022_5617_MOESM1_ESM.docx]

**S1 Table. Apparent density of tsetse flies in Ethiopia by zone.**

Data collection period: 2010–2019.

Gp: *G. pallidipes*. Gms: *G. morsitans submorsitans*. Gff: *G. fuscipes fuscipes*. Gt: *G. tachinoides*. Gl: *G. longipennis.*

| **Region** | **Zone** | **Trapping locations [n]** | **Trapping events [n]** | **Trapping**  **Intensity**  **[trap days]** | **Tsetse flies [flies/trap/day]** | | | | | |
| --- | --- | --- | --- | --- | --- | --- | --- | --- | --- | --- |
|  |  |  |  |  | ***Gp*** | ***Gms*** | ***Gff*** | ***Gt*** | ***Gl*** | **TOTAL** |
| Amhara | Awi | 688 | 693 | 1402 | 0 | 0 | 0 | 1.78 | 0 | 1.78 |
| Amhara | Central Gondar | 59 | 59 | 118 | 0 | 0 | 0 | 0 | 0 | 0.00 |
| Amhara | East Gojam | 30 | 30 | 60 | 0 | 0 | 0 | 0.20 | 0 | 0.20 |
| Amhara | West Gojam | 411 | 416 | 850 | 0 | 0.08 | 0 | 4.64 | 0 | 4.72 |
| Amhara | West Gondar | 189 | 190 | 388 | 0 | 0 | 0 | 0.75 | 0 | 0.75 |
| Benshangul Gumuz | Assosa | 479 | 528 | 1282 | 0 | 3.70 | 0 | 1.03 | 0 | 4.73 |
| Benshangul Gumuz | Metekel | 2183 | 2243 | 4707 | 0 | 0.07 | 0 | 2.74 | 0 | 2.81 |
| Gambela | Agnewak | 263 | 263 | 561 | 0.02 | 0.04 | 0.04 | 0.05 | 0 | 0.15 |
| Gambela | Itang Special | 40 | 40 | 80 | 0 | 0 | 0 | 0 | 0 | 0.00 |
| Gambela | Nuwer | 154 | 154 | 436 | 0 | 0 | 0.64 | 2.79 | 0 | 3.44 |
| Oromia | Borena | 50 | 95 | 310 | 1.24 | 0 | 0 | 0 | 0 | 1.24 |
| Oromia | Buno Bedele | 1157 | 1235 | 3217 | 0.37 | 1.18 | 0.07 | 3.02 | 0 | 4.63 |
| Oromia | East Wellega | 577 | 586 | 1421 | 0.12 | 0.19 | 0 | 1.97 | 0 | 2.28 |
| Oromia | Horo Guduru | 352 | 352 | 763 | 0 | 0.77 | 0 | 0.31 | 0 | 1.08 |
| Oromia | Ilu Aba Bora | 1350 | 1401 | 4672 | 1.46 | 0.93 | 0.58 | 0.68 | 0 | 3.66 |
| Oromia | Jimma | 902 | 967 | 2609 | 0.41 | 1.09 | 0.42 | 0.39 | 0 | 2.31 |
| Oromia | Kelem Welega | 765 | 823 | 2227 | 0.89 | 0.46 | 0.44 | 0.13 | 0 | 1.92 |
| Oromia | West Guji | 342 | 806 | 2478 | 1.39 | 0 | 0 | 0 | 0 | 1.39 |
| Oromia | West Shewa | 101 | 102 | 282 | 0.34 | 0.66 | 0.35 | 0 | 0 | 1.36 |
| Oromia | West Wellega | 283 | 320 | 1154 | 0.23 | 0.73 | 0.29 | 0.59 | 0 | 1.83 |
| SNNP | Ale special woreda | 11 | 13 | 39 | 0 | 0 | 0 | 0 | 0 | 0.00 |
| SNNP | Amaro special woreda | 140 | 282 | 891 | 0.30 | 0 | 0 | 0 | 0 | 0.30 |
| SNNP | Basketo special wereda | 27 | 30 | 111 | 0.38 | 0 | 0 | 0 | 0 | 0.38 |
| SNNP | Bench_Sheko | 10 | 10 | 39 | 1.08 | 0 | 0 | 0 | 0 | 1.08 |
| SNNP | Burji special woreda | 72 | 115 | 325 | 0.65 | 0 | 0 | 0 | 0 | 0.65 |
| SNNP | Dawuro | 173 | 228 | 622 | 3.13 | 0 | 0.42 | 0 | 0 | 3.56 |
| SNNP | Derashe special woreda | 25 | 25 | 75 | 0 | 0 | 0 | 0 | 0 | 0.00 |
| SNNP | Gamo | 1885 | 2405 | 7273 | 5.24 | 0 | 0.30 | 0 | 0 | 5.54 |
| SNNP | Gofa | 185 | 301 | 960 | 3.33 | 0 | 0.02 | 0 | 0 | 3.35 |
| SNNP | Gurage | 77 | 130 | 332 | 0.52 | 0 | 0 | 0 | 3.01 × 10^-3^ | 0.52 |
| SNNP | Hadiya | 64 | 67 | 206 | 0.07 | 0 | 0 | 0 | 0 | 0.07 |
| SNNP | Kambata Tembaro | 21 | 24 | 62 | 0.23 | 0 | 0 | 0 | 0 | 0.23 |
| SNNP | Kefa | 60 | 60 | 170 | 0.61 | 0 | 0 | 0 | 0 | 0.61 |
| SNNP | Konso | 88 | 164 | 492 | 2.22 | 0 | 0 | 0 | 0 | 2.22 |
| SNNP | Konta special wereda | 45 | 69 | 221 | 7.27 | 0 | 0.11 | 0 | 0 | 7.38 |
| SNNP | Sidama | 242 | 342 | 1036 | 3.04 | 0 | 0 | 0 | 0 | 3.04 |
| SNNP | South Omo | 231 | 364 | 1152 | 5.09 | 0 | 2.60 × 10^-3^ | 0 | 0 | 5.09 |
| SNNP | West Omo | 65 | 65 | 200 | 0.44 | 0 | 0.75 | 0 | 0 | 1.19 |
| SNNP | Wolayita | 702 | 868 | 2597 | 1.59 | 0 | 0.09 | 0 | 0 | 1.69 |
| TOTAL |  | 14,498 | 16,865 | 45,820 | 1.65 | 0.42 | 0.19 | 0.88 | 2.18 × 10^-5^ | 3.13 |
