# Supplementary material for: The national atlas of tsetse flies and African animal trypanosomosis in Ethiopia
Source: Parasit Vectors. 2022 Dec 28;15:491. doi: 10.1186/s13071-022-05617-9 (PMC9798648; doi:10.1186/s13071-022-05617-9)
Supplement: Supplementary file 2 — Additional file 2: S2. Apparent density of tsetse flies in Ethiopia by district (woreda). Data collection period: 2010–2019. [file 13071_2022_5617_MOESM2_ESM.docx]

**S2 Table. Apparent density of tsetse flies in Ethiopia by district (*woreda*).**

Data collection period: 2010–2019.

Gp: *G. pallidipes*. Gms: *G. morsitans submorsitans*. Gff: *G. fuscipes fuscipes*. Gt: *G. tachinoides*. Gl: *G. longipennis.*

| **Region** | **Zone** | **Woreda** | **Trapping locations [n]** | **Trapping events [n]** | **Trapping intensity [trap days]** | **Tsetse flies**  **[flies/trap/day]** | | | | | |
| --- | --- | --- | --- | --- | --- | --- | --- | --- | --- | --- | --- |
|  |  |  |  |  |  | ***Gp*** | ***Gms*** | ***Gff*** | ***Gt*** | ***Gl*** | **TOTAL** |
| Amhara | Awi | Ankasha Guagusa | 117 | 117 | 234 | 0 | 0 | 0 | 0.55 | 0 | 0.55 |
| Amhara | Awi | Guangua | 69 | 69 | 138 | 0 | 0 | 0 | 0 | 0 | 0.00 |
| Amhara | Awi | Jawi | 502 | 507 | 1030 | 0 | 0 | 0 | 2.30 | 0 | 2.30 |
| Amhara | Central Gondar | Alfa | 59 | 59 | 118 | 0 | 0 | 0 | 0 | 0 | 0.00 |
| Amhara | East Gojam | Deberelias | 30 | 30 | 60 | 0 | 0 | 0 | 0.20 | 0 | 0.20 |
| Amhara | West Gojam | Bure (AM) | 69 | 69 | 138 | 0 | 0 | 0 | 0 | 0 | 0.00 |
| Amhara | West Gojam | Dembecha | 83 | 83 | 184 | 0 | 0.36 | 0 | 0.13 | 0 | 0.49 |
| Amhara | West Gojam | Jabitehenan | 23 | 23 | 46 | 0 | 0 | 0 | 0.46 | 0 | 0.46 |
| Amhara | West Gojam | North Achefer | 48 | 48 | 96 | 0 | 0 | 0 | 0.05 | 0 | 0.05 |
| Amhara | West Gojam | South Achefer | 166 | 171 | 342 | 0 | 0 | 0 | 11.38 | 0 | 11.38 |
| Amhara | West Gojam | Wemberma | 22 | 22 | 44 | 0 | 0 | 0 | 0 | 0 | 0.00 |
| Amhara | West Gondar | Quara | 189 | 190 | 388 | 0 | 0 | 0 | 0.75 | 0 | 0.75 |
| Benshangul Gumuz | Assosa | Assosa | 222 | 222 | 444 | 0 | 5.42 | 0 | 0 | 0 | 5.42 |
| Benshangul Gumuz | Assosa | Bambasi | 187 | 236 | 624 | 0 | 2.80 | 0 | 1.24 | 0 | 4.04 |
| Benshangul Gumuz | Assosa | Oda Bildigilu | 70 | 70 | 214 | 0 | 2.76 | 0 | 2.57 | 0 | 5.33 |
| Benshangul Gumuz | Metekel | Bulen | 233 | 234 | 539 | 0 | 0.02 | 0 | 0.85 | 0 | 0.87 |
| Benshangul Gumuz | Metekel | Dangura | 577 | 606 | 1278 | 0 | 0.04 | 0 | 1.98 | 0 | 2.03 |
| Benshangul Gumuz | Metekel | Debati | 161 | 170 | 344 | 0 | 0 | 0 | 1.06 | 0 | 1.06 |
| Benshangul Gumuz | Metekel | Guba | 108 | 108 | 216 | 0 | 0 | 0 | 2.41 | 0 | 2.41 |
| Benshangul Gumuz | Metekel | Mandura | 465 | 481 | 1002 | 0 | 0 | 0 | 1.44 | 0 | 1.44 |
| Benshangul Gumuz | Metekel | Pawe | 558 | 563 | 1136 | 0 | 0 | 0 | 5.89 | 0 | 5.89 |
| Benshangul Gumuz | Metekel | Wenbera | 81 | 81 | 192 | 0 | 1.26 | 0 | 4.66 | 0 | 5.92 |
| Gambela | Agnewak | Abobo | 147 | 147 | 296 | 0.03 | 0.05 | 0.05 | 0.01 | 0 | 0.15 |
| Gambela | Agnewak | Gambela Zuria | 100 | 100 | 233 | 0 | 0.04 | 0.01 | 0.10 | 0 | 0.15 |
| Gambela | Agnewak | Gog | 16 | 16 | 32 | 0 | 0 | 0.13 | 0 | 0 | 0.13 |
| Gambela | Itang Special | Itang | 40 | 40 | 80 | 0 | 0 | 0 | 0 | 0 | 0.00 |
| Gambela | Nuwer | Jikaw | 40 | 40 | 120 | 0 | 0 | 0 | 0 | 0 | 0.00 |
| Gambela | Nuwer | Lare | 84 | 84 | 256 | 0 | 0 | 1.10 | 3.71 | 0 | 4.81 |
| Gambela | Nuwer | Wantawo | 30 | 30 | 60 | 0 | 0 | 0 | 4.45 | 0 | 4.45 |
| Oromia | Borena | Ale Woya | 10 | 10 | 60 | 0.87 | 0 | 0 | 0 | 0 | 0.87 |
| Oromia | Borena | Teltele | 30 | 75 | 220 | 1.51 | 0 | 0 | 0 | 0 | 1.51 |
| Oromia | Borena | Yabello | 10 | 10 | 30 | 0 | 0 | 0 | 0 | 0 | 0.00 |
| Oromia | Buno Bedele | Bedele Zuria | 281 | 305 | 616 | 1.01 | 0 | 0 | 3.43 | 0 | 4.43 |
| Oromia | Buno Bedele | Boricha (Bunobedele) | 113 | 114 | 335 | 0 | 4.51 | 0 | 6.34 | 0 | 10.85 |
| Oromia | Buno Bedele | Chewaka | 162 | 200 | 624 | 0 | 1.90 | 0 | 2.43 | 0 | 4.33 |
| Oromia | Buno Bedele | Chora (Bedele) | 151 | 165 | 448 | 0.81 | 0.48 | 0.47 | 0.27 | 0 | 2.03 |
| Oromia | Buno Bedele | Dabo Hana | 147 | 148 | 354 | 0 | 1.34 | 0 | 7.32 | 0 | 8.66 |
| Oromia | Buno Bedele | Didessa | 120 | 120 | 240 | 0 | 0 | 0.07 | 1.81 | 0 | 1.88 |
| Oromia | Buno Bedele | Gechi | 143 | 143 | 488 | 0.41 | 0.76 | 0 | 1.54 | 0 | 2.71 |
| Oromia | Buno Bedele | Meko | 40 | 40 | 112 | 0 | 0.23 | 0 | 0.59 | 0 | 0.82 |
| Oromia | East Wellega | Diga | 61 | 61 | 122 | 0 | 0 | 0 | 0.98 | 0 | 0.98 |
| Oromia | East Wellega | Gida Ayana | 52 | 52 | 108 | 0 | 0 | 0 | 1.09 | 0 | 1.09 |
| Oromia | East Wellega | Gobu Seyo | 29 | 29 | 75 | 0 | 0.79 | 0 | 0 | 0 | 0.79 |
| Oromia | East Wellega | Guto Gida | 86 | 86 | 204 | 0 | 0 | 0 | 7.37 | 0 | 7.37 |
| Oromia | East Wellega | Jimma Arjo | 53 | 62 | 158 | 0 | 0 | 0 | 3.82 | 0 | 3.82 |
| Oromia | East Wellega | Leqa Dulacha | 36 | 36 | 108 | 0 | 0 | 0 | 1.53 | 0 | 1.53 |
| Oromia | East Wellega | Nunu Kumba | 58 | 58 | 120 | 0 | 0.14 | 0 | 0.55 | 0 | 0.69 |
| Oromia | East Wellega | Sasiga | 57 | 57 | 180 | 0 | 0.57 | 0 | 0.43 | 0 | 1.01 |
| Oromia | East Wellega | Sibusire | 49 | 49 | 98 | 0.08 | 0.09 | 0 | 0 | 0 | 0.17 |
| Oromia | East Wellega | Wama Hagelo | 60 | 60 | 162 | 0 | 0.19 | 0 | 0.89 | 0 | 1.07 |
| Oromia | East Wellega | Wayu Tuqa | 36 | 36 | 86 | 1.84 | 0.67 | 0 | 0 | 0 | 2.51 |
| Oromia | Horo Guduru | Abay Chomen | 68 | 68 | 142 | 0 | 0.44 | 0 | 0.21 | 0 | 0.65 |
| Oromia | Horo Guduru | Abe Dongoro | 97 | 97 | 194 | 0 | 1.37 | 0 | 0.22 | 0 | 1.59 |
| Oromia | Horo Guduru | Amuru | 92 | 92 | 225 | 0 | 0.68 | 0 | 0.40 | 0 | 1.07 |
| Oromia | Horo Guduru | Jarte Jardega | 95 | 95 | 202 | 0 | 0.52 | 0 | 0.38 | 0 | 0.90 |
| Oromia | Ilu Aba Bora | Alge Sachi | 126 | 127 | 520 | 0.19 | 0.72 | 0.65 | 0.70 | 0 | 2.26 |
| Oromia | Ilu Aba Bora | Bilonopa | 118 | 118 | 262 | 0.72 | 0.74 | 1.01 | 1.11 | 0 | 3.57 |
| Oromia | Ilu Aba Bora | Bure (OR) | 376 | 399 | 1376 | 2.71 | 1.05 | 0.38 | 0.37 | 0 | 4.50 |
| Oromia | Ilu Aba Bora | Darimu | 230 | 245 | 844 | 2.89 | 2.30 | 0.72 | 0.52 | 0 | 6.42 |
| Oromia | Ilu Aba Bora | Doreni | 106 | 107 | 408 | 0.68 | 0 | 0.96 | 1.52 | 0 | 3.16 |
| Oromia | Ilu Aba Bora | Halu/Huka | 100 | 110 | 264 | 0.17 | 0.46 | 0.06 | 0.28 | 0 | 0.98 |
| Oromia | Ilu Aba Bora | Hurumu | 88 | 89 | 302 | 0 | 0.11 | 0.61 | 0.64 | 0 | 1.36 |
| Oromia | Ilu Aba Bora | Metu | 99 | 99 | 270 | 0.23 | 0.16 | 0.34 | 0.37 | 0 | 1.10 |
| Oromia | Ilu Aba Bora | Yayo | 107 | 107 | 426 | 0 | 0.52 | 0.72 | 1.38 | 0 | 2.61 |
| Oromia | Jimma | Boter Tolay | 140 | 140 | 388 | 0.26 | 0.09 | 1.12 | 1.29 | 0 | 2.76 |
| Oromia | Jimma | Chora (Jimma) | 16 | 16 | 38 | 0 | 1.21 | 1.18 | 0 | 0 | 2.39 |
| Oromia | Jimma | Dedo | 49 | 49 | 174 | 0 | 3.39 | 0 | 0 | 0 | 3.39 |
| Oromia | Jimma | Goma | 122 | 154 | 362 | 0 | 1.07 | 0.27 | 0.14 | 0 | 1.48 |
| Oromia | Jimma | Gumay | 70 | 70 | 148 | 0 | 0.05 | 0 | 0.18 | 0 | 0.22 |
| Oromia | Jimma | Limu kosa | 92 | 94 | 200 | 0 | 0 | 0.15 | 1.41 | 0 | 1.55 |
| Oromia | Jimma | Limu Seka | 120 | 120 | 242 | 0 | 0 | 0 | 0.68 | 0 | 0.68 |
| Oromia | Jimma | Nono Benja | 8 | 8 | 16 | 0 | 0 | 0 | 0 | 0 | 0.00 |
| Oromia | Jimma | Omo Beyam | 25 | 25 | 123 | 1.49 | 2.50 | 1.43 | 0 | 0 | 5.42 |
| Oromia | Jimma | Sekoru | 107 | 138 | 456 | 1.27 | 2.20 | 0.19 | 0 | 0 | 3.66 |
| Oromia | Jimma | Shebe Sombo | 116 | 116 | 352 | 0.56 | 1.02 | 0.32 | 0 | 0 | 1.90 |
| Oromia | Jimma | Tiro Afeta | 37 | 37 | 110 | 0 | 1.09 | 1.01 | 0 | 0 | 2.10 |
| Oromia | Kelem Welega | Dale Sadi | 182 | 211 | 699 | 1.10 | 0.35 | 0.51 | 0.38 | 0 | 2.33 |
| Oromia | Kelem Welega | Dale Wabera/Sedi Chenka | 143 | 146 | 466 | 1.40 | 0.14 | 0.47 | 0 | 0 | 2.01 |
| Oromia | Kelem Welega | Hawa Gelan | 296 | 322 | 746 | 0.73 | 0.05 | 0.13 | 0.03 | 0 | 0.95 |
| Oromia | Kelem Welega | Lalo Kile | 144 | 144 | 316 | 0.06 | 2.13 | 0.95 | 0.01 | 0 | 3.15 |
| Oromia | West Guji | Abaya | 257 | 595 | 1815 | 1.36 | 0 | 0 | 0 | 0 | 1.36 |
| Oromia | West Guji | Gelana | 70 | 176 | 558 | 1.73 | 0 | 0 | 0 | 0 | 1.73 |
| Oromia | West Guji | Suro Bariguda | 15 | 35 | 105 | 0 | 0 | 0 | 0 | 0 | 0.00 |
| Oromia | West Shewa | Bako Tibe | 24 | 24 | 48 | 0 | 0 | 0 | 0 | 0 | 0.00 |
| Oromia | West Shewa | Dano | 42 | 42 | 92 | 0.16 | 0.10 | 0.52 | 0 | 0 | 0.78 |
| Oromia | West Shewa | Nono | 35 | 36 | 142 | 0.58 | 1.25 | 0.37 | 0 | 0 | 2.20 |
| Oromia | West Wellega | Ayira | 83 | 84 | 202 | 0 | 0.53 | 0.08 | 0.17 | 0 | 0.78 |
| Oromia | West Wellega | Guliso | 40 | 40 | 130 | 0.11 | 0.52 | 0.25 | 0.48 | 0 | 1.37 |
| Oromia | West Wellega | Haru | 25 | 48 | 178 | 0 | 0.58 | 0.52 | 0.69 | 0 | 1.79 |
| Oromia | West Wellega | Nole Kaba | 101 | 111 | 414 | 0.18 | 0.63 | 0.32 | 0.70 | 0 | 1.83 |
| Oromia | West Wellega | Yubdo | 34 | 37 | 230 | 0.79 | 1.31 | 0.23 | 0.72 | 0 | 3.05 |
| SNNP | Ale special woreda | Ale | 11 | 13 | 39 | 0 | 0 | 0 | 0 | 0 | 0.00 |
| SNNP | Amaro special woreda | Amaro | 140 | 282 | 891 | 0.30 | 0 | 0 | 0 | 0 | 0.30 |
| SNNP | Basketo special wereda | Basketo special wereda | 27 | 30 | 111 | 0.38 | 0 | 0 | 0 | 0 | 0.38 |
| SNNP | Bench_Sheko | Debub Bench | 5 | 5 | 24 | 1.08 | 0 | 0 | 0 | 0 | 1.08 |
| SNNP | Bench_Sheko | Gura Ferda | 5 | 5 | 15 | 1.07 | 0 | 0 | 0 | 0 | 1.07 |
| SNNP | Burji special woreda | Burji | 72 | 115 | 325 | 0.65 | 0 | 0 | 0 | 0 | 0.65 |
| SNNP | Dawuro | Disa | 10 | 10 | 25 | 2.80 | 0 | 0 | 0 | 0 | 2.80 |
| SNNP | Dawuro | Esara | 5 | 5 | 24 | 0.33 | 0 | 0.33 | 0 | 0 | 0.67 |
| SNNP | Dawuro | Gena Bosa | 40 | 75 | 192 | 5.96 | 0 | 0.09 | 0 | 0 | 6.05 |
| SNNP | Dawuro | Loma | 86 | 106 | 287 | 1.84 | 0 | 0.63 | 0 | 0 | 2.47 |
| SNNP | Dawuro | Mareka | 5 | 5 | 15 | 0.20 | 0 | 0 | 0 | 0 | 0.20 |
| SNNP | Dawuro | Tercha | 22 | 22 | 64 | 3.00 | 0 | 0.91 | 0 | 0 | 3.91 |
| SNNP | Dawuro | Tocha | 5 | 5 | 15 | 0.20 | 0 | 0 | 0 | 0 | 0.20 |
| SNNP | Derashe special woreda | Derashe | 25 | 25 | 75 | 0 | 0 | 0 | 0 | 0 | 0.00 |
| SNNP | Gamo | Arbaminch Zurya | 342 | 441 | 1313 | 6.23 | 0 | 0 | 0 | 0 | 6.23 |
| SNNP | Gamo | Boreda | 136 | 146 | 444 | 0 | 0 | 2.25 × 10^-3^ | 0 | 0 | 0.00 |
| SNNP | Gamo | Daramalo | 49 | 122 | 367 | 7.20 | 0 | 0 | 0 | 0 | 7.20 |
| SNNP | Gamo | Kemba | 68 | 100 | 311 | 4.41 | 0 | 0 | 0 | 0 | 4.41 |
| SNNP | Gamo | Kucha | 481 | 632 | 1928 | 0.67 | 0 | 1.14 | 0 | 0 | 1.82 |
| SNNP | Gamo | Mirab Abaya | 809 | 964 | 2910 | 8.45 | 0 | 0 | 0 | 0 | 8.45 |
| SNNP | Gofa | Deniba Gofa | 48 | 71 | 234 | 8.26 | 0 | 0 | 0 | 0 | 8.26 |
| SNNP | Gofa | Melekoza | 36 | 40 | 173 | 1.06 | 0 | 0.13 | 0 | 0 | 1.19 |
| SNNP | Gofa | Ouba Debretsehay | 8 | 30 | 90 | 0.50 | 0 | 0 | 0 | 0 | 0.50 |
| SNNP | Gofa | Zala | 93 | 160 | 463 | 2.23 | 0 | 0 | 0 | 0 | 2.23 |
| SNNP | Gurage | Abeshige | 30 | 60 | 162 | 0.99 | 0 | 0 | 0 | 6.17 × 10^-3^ | 0.99 |
| SNNP | Gurage | Cheha | 10 | 10 | 30 | 0.03 | 0 | 0 | 0 | 0 | 0.03 |
| SNNP | Gurage | Enemor Ener | 37 | 60 | 140 | 0.08 | 0 | 0 | 0 | 0 | 0.08 |
| SNNP | Hadiya | Ameka | 11 | 11 | 33 | 0.06 | 0 | 0 | 0 | 0 | 0.06 |
| SNNP | Hadiya | Gibe | 10 | 10 | 30 | 0 | 0 | 0 | 0 | 0 | 0.00 |
| SNNP | Hadiya | Gombora | 6 | 6 | 33 | 0.06 | 0 | 0 | 0 | 0 | 0.06 |
| SNNP | Hadiya | Soro | 37 | 40 | 110 | 0.09 | 0 | 0 | 0 | 0 | 0.09 |
| SNNP | Kambata Tembaro | Tembaro | 21 | 24 | 62 | 0.23 | 0 | 0 | 0 | 0 | 0.23 |
| SNNP | Kefa | Bita | 20 | 20 | 50 | 0.08 | 0 | 0 | 0 | 0 | 0.08 |
| SNNP | Kefa | Chena | 10 | 10 | 30 | 0 | 0 | 0 | 0 | 0 | 0.00 |
| SNNP | Kefa | Cheta | 10 | 10 | 30 | 0.03 | 0 | 0 | 0 | 0 | 0.03 |
| SNNP | Kefa | Gimbo | 20 | 20 | 60 | 1.65 | 0 | 0 | 0 | 0 | 1.65 |
| SNNP | Konso | Konso | 88 | 164 | 492 | 2.22 | 0 | 0 | 0 | 0 | 2.22 |
| SNNP | Konta special wereda | Konta special wereda | 45 | 69 | 221 | 7.27 | 0 | 0.11 | 0 | 0 | 7.38 |
| SNNP | Sidama | Dara | 27 | 37 | 111 | 0 | 0 | 0 | 0 | 0 | 0.00 |
| SNNP | Sidama | Darara | 10 | 10 | 30 | 0 | 0 | 0 | 0 | 0 | 0.00 |
| SNNP | Sidama | Loka Abaya | 205 | 295 | 895 | 3.52 | 0 | 0 | 0 | 0 | 3.52 |
| SNNP | South Omo | Bena Tsemay | 58 | 135 | 393 | 0.07 | 0 | 0 | 0 | 0 | 0.07 |
| SNNP | South Omo | Dasenech | 16 | 20 | 75 | 1.20 | 0 | 0 | 0 | 0 | 1.20 |
| SNNP | South Omo | Gnangatom | 15 | 15 | 54 | 0.52 | 0 | 0 | 0 | 0 | 0.52 |
| SNNP | South Omo | Hamer | 49 | 50 | 165 | 0.35 | 0 | 0 | 0 | 0 | 0.35 |
| SNNP | South Omo | Male | 31 | 34 | 102 | 0 | 0 | 0 | 0 | 0 | 0.00 |
| SNNP | South Omo | Selamago | 32 | 70 | 213 | 25.37 | 0 | 0 | 0 | 0 | 25.37 |
| SNNP | South Omo | South Ari | 30 | 40 | 150 | 1.73 | 0 | 0.02 | 0 | 0 | 1.75 |
| SNNP | West Omo | Bero | 20 | 20 | 60 | 0.23 | 0 | 0 | 0 | 0 | 0.23 |
| SNNP | West Omo | Maji | 20 | 20 | 60 | 0.37 | 0 | 0 | 0 | 0 | 0.37 |
| SNNP | West Omo | Menit Goldia | 25 | 25 | 80 | 0.64 | 0 | 1.88 | 0 | 0 | 2.51 |
| SNNP | Wolayita | Abela Abaya | 127 | 150 | 450 | 0.97 | 0 | 0 | 0 | 0 | 0.97 |
| SNNP | Wolayita | Boloso Bombe | 15 | 15 | 45 | 0 | 0 | 0 | 0 | 0 | 0.00 |
| SNNP | Wolayita | Damot woyde | 85 | 85 | 255 | 0.27 | 0 | 0 | 0 | 0 | 0.27 |
| SNNP | Wolayita | Duguna Fango | 151 | 174 | 536 | 0.79 | 0 | 0 | 0 | 0 | 0.79 |
| SNNP | Wolayita | Hobicha Abaya | 140 | 204 | 612 | 4.80 | 0 | 0 | 0 | 0 | 4.80 |
| SNNP | Wolayita | Humbo | 15 | 15 | 45 | 0 | 0 | 0 | 0 | 0 | 0.00 |
| SNNP | Wolayita | Kindo Didaye | 39 | 52 | 143 | 1.73 | 0 | 0.14 | 0 | 0 | 1.87 |
| SNNP | Wolayita | Kindo Koysha | 10 | 15 | 60 | 0.43 | 0 | 0 | 0 | 0 | 0.43 |
| SNNP | Wolayita | Offa | 120 | 158 | 451 | 0 | 0 | 0.49 | 0 | 0 | 0.49 |
| TOTAL |  |  | 14,498 | 16,865 | 45,820 | 1.65 | 0.42 | 0.19 | 0.88 | 2.18 × 10^-5^ | 3.13 |
