# Supplementary material for: The national atlas of tsetse flies and African animal trypanosomosis in Ethiopia
Source: Parasit Vectors. 2022 Dec 28;15:491. doi: 10.1186/s13071-022-05617-9 (PMC9798648; doi:10.1186/s13071-022-05617-9)

Glossina pallidipes (2010 - 2019)

The national atlas of tsetse and African animal trypanosomosis in Ethiopia

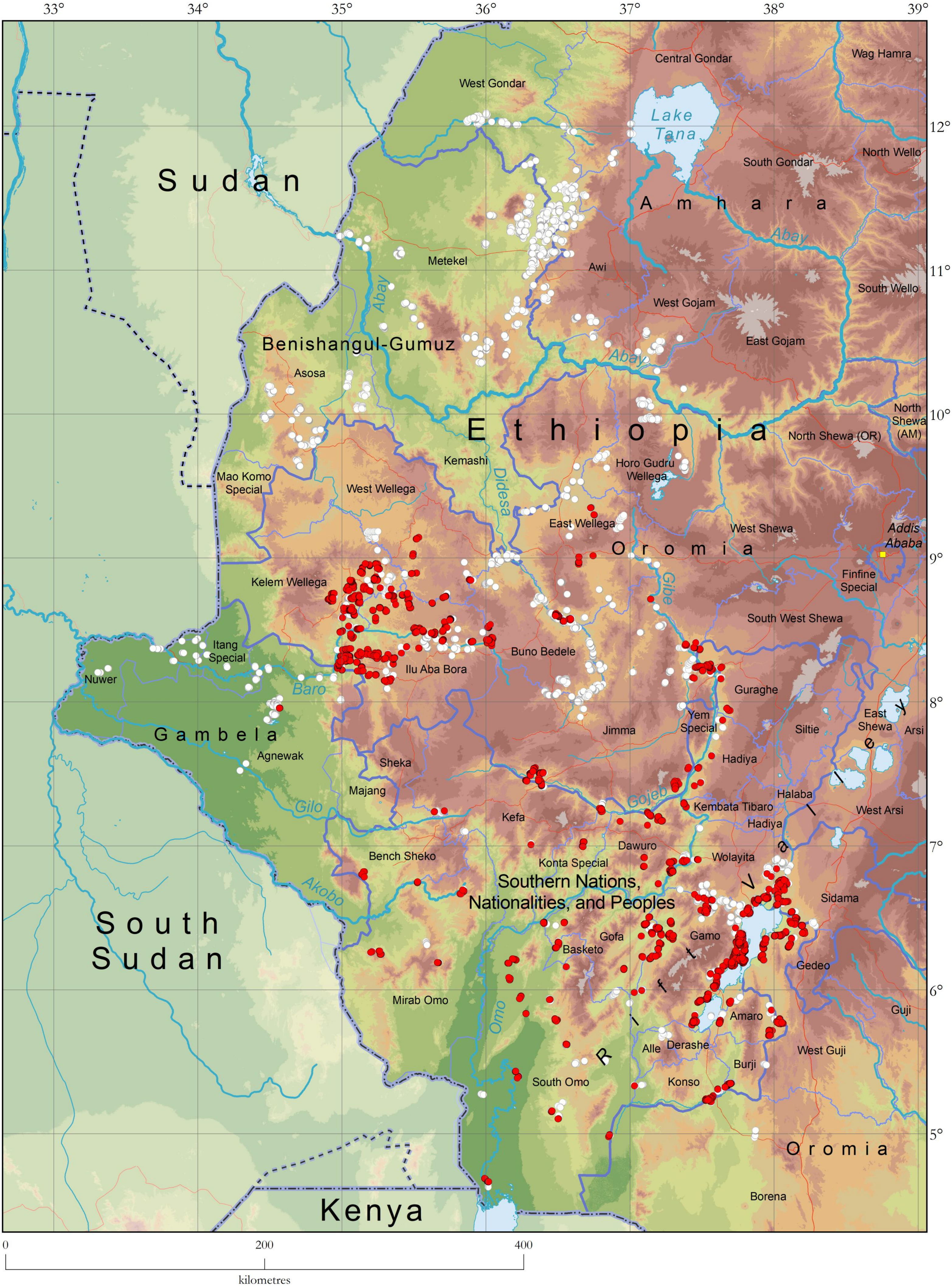

Glossina pallidipes (2010 - 2019)

The national atlas of tsetse and African animal trypanosomosis in Ethiopia

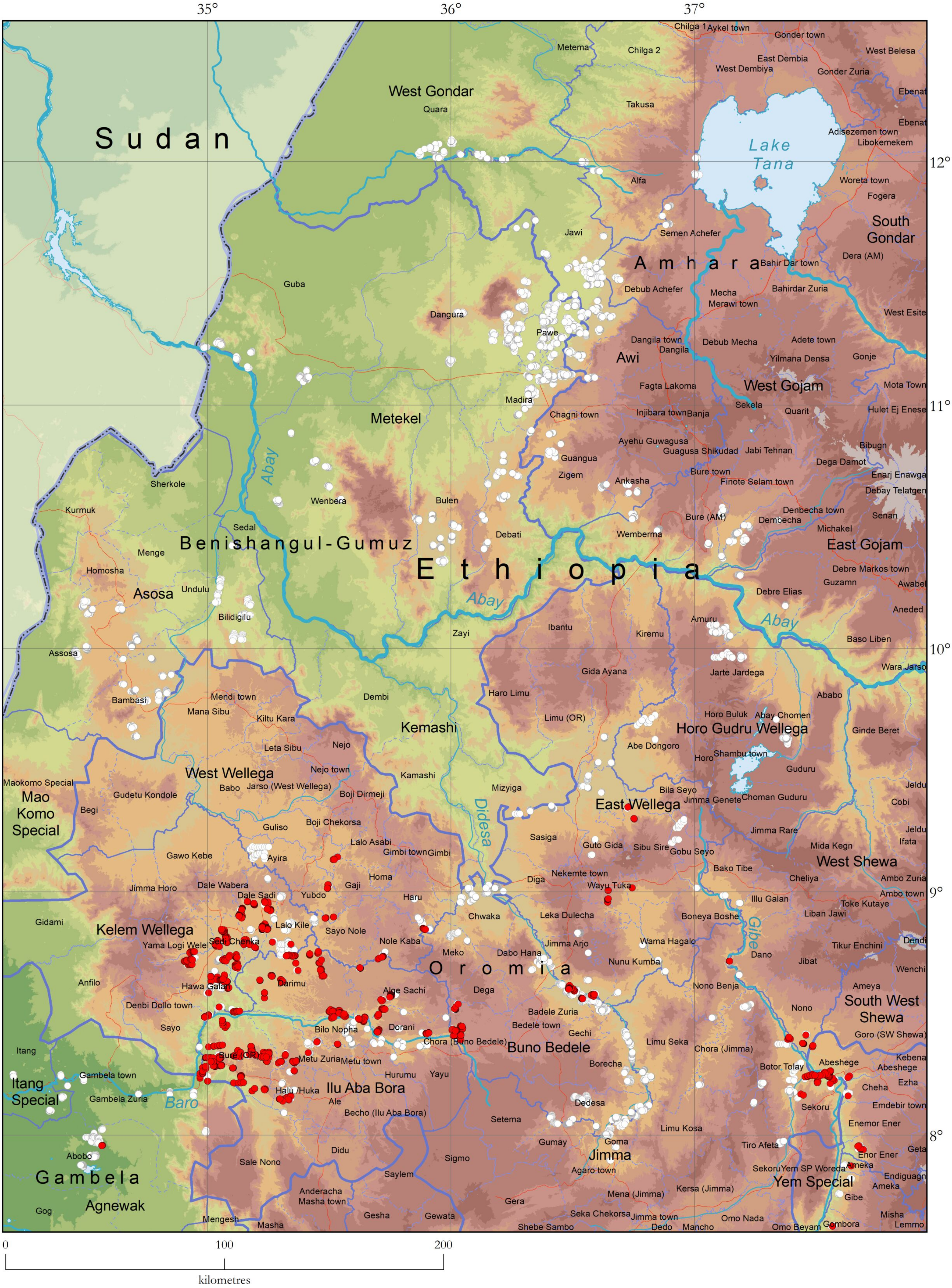

Glossina pallidipes (2010 - 2019)

The national atlas of tsetse and African animal trypanosomosis in Ethiopia

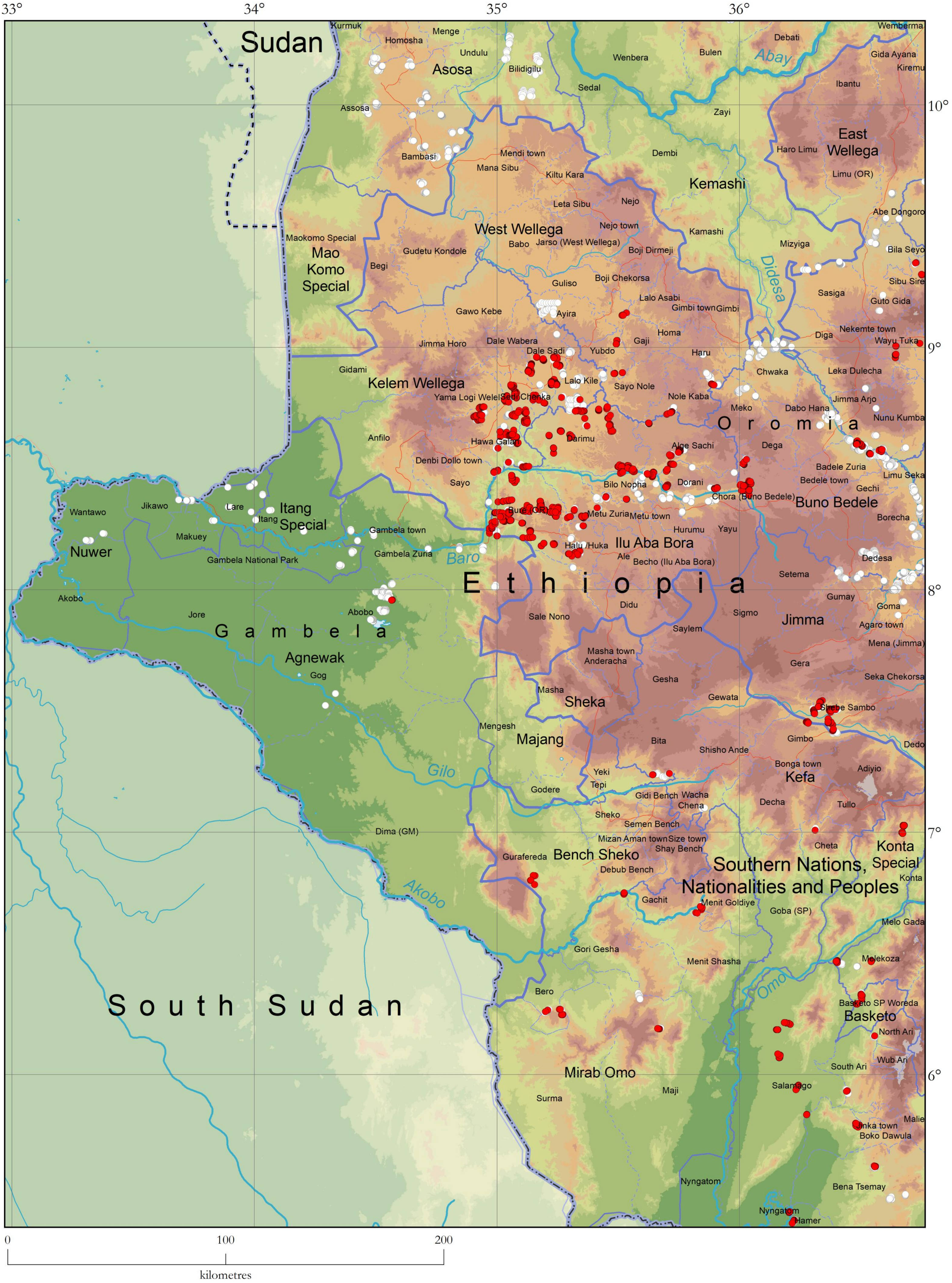

Glossina pallidipes (2010 - 2019)

The national atlas of tsetse and African animal trypanosomosis in Ethiopia

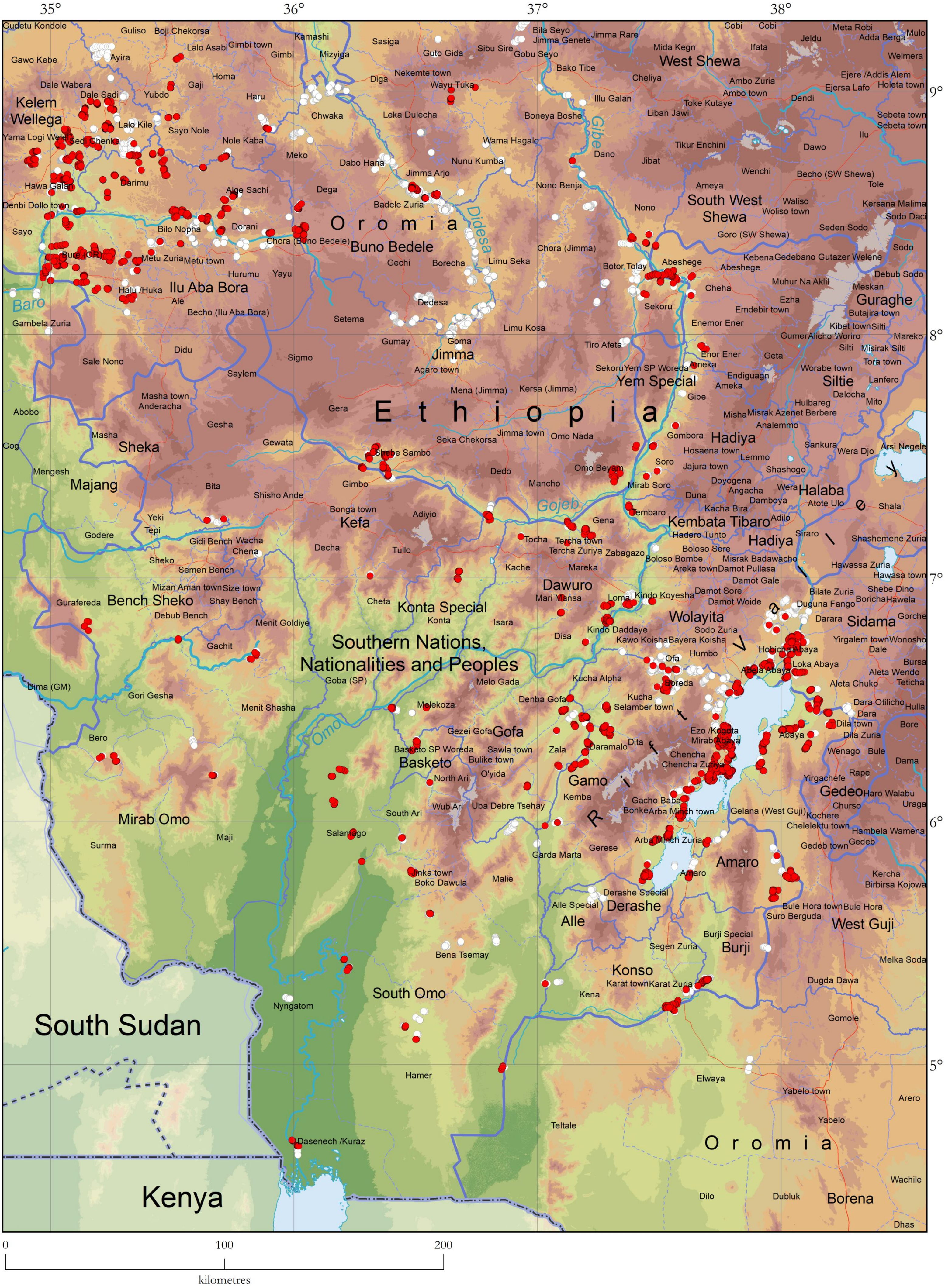

Glossina morsitans submorsitans (2010 - 2019)

The national atlas of tsetse and African animal trypanosomosis in Ethiopia

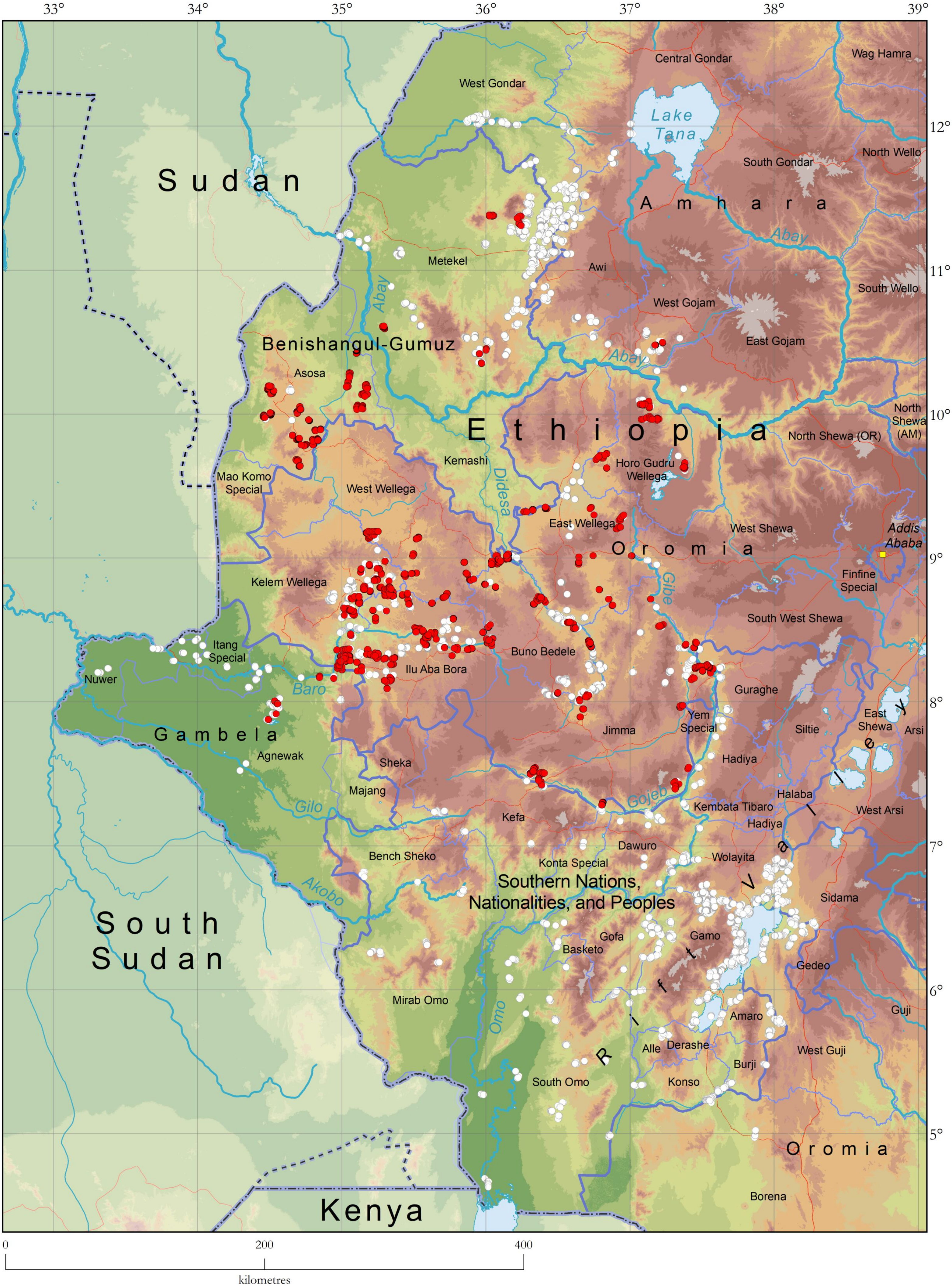

Glossina morsitans submorsitans (2010 - 2019)

The national atlas of tsetse and African animal trypanosomosis in Ethiopia

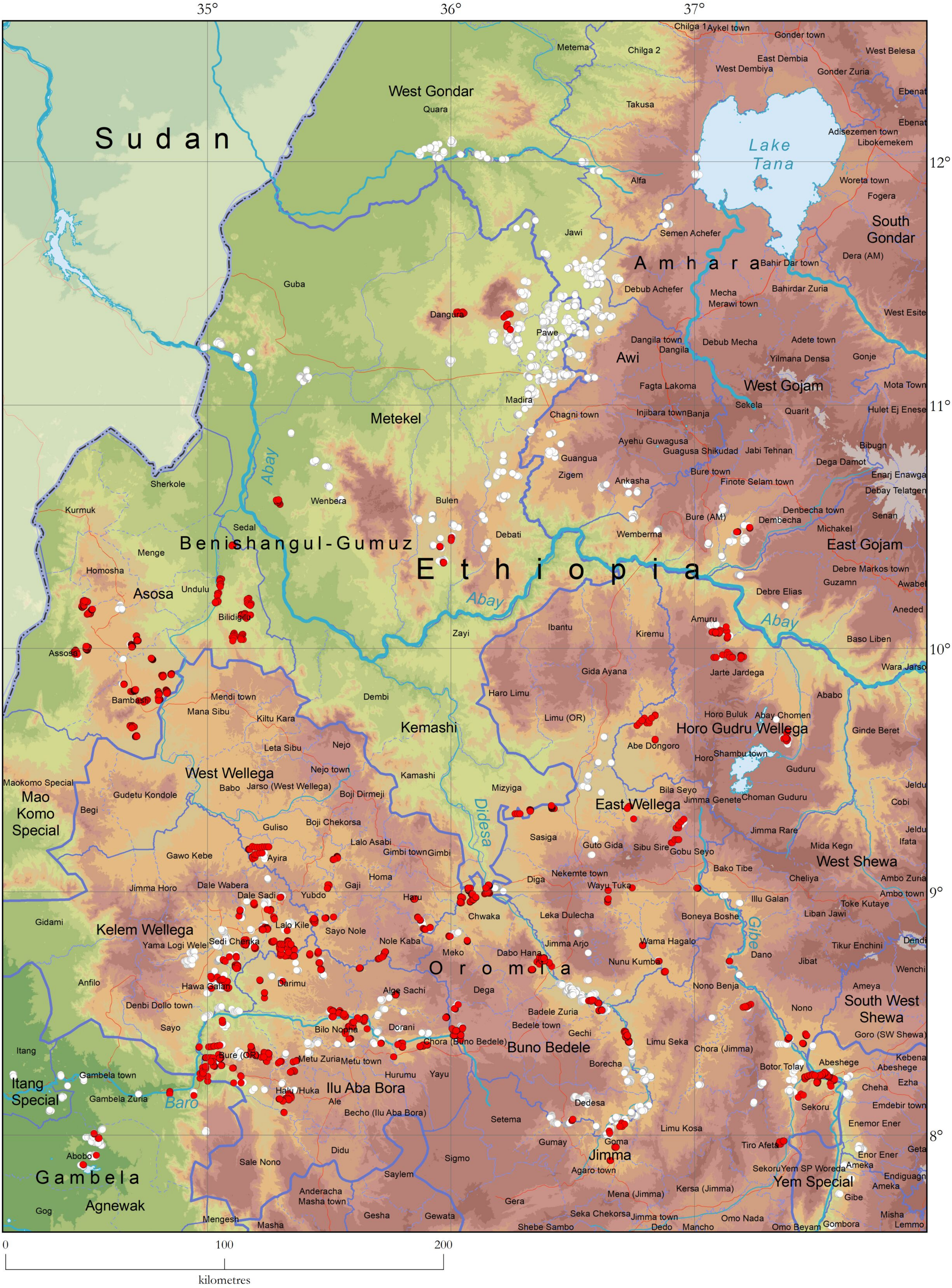

Glossina morsitans submorsitans (2010 - 2019)

The national atlas of tsetse and African animal trypanosomosis in Ethiopia

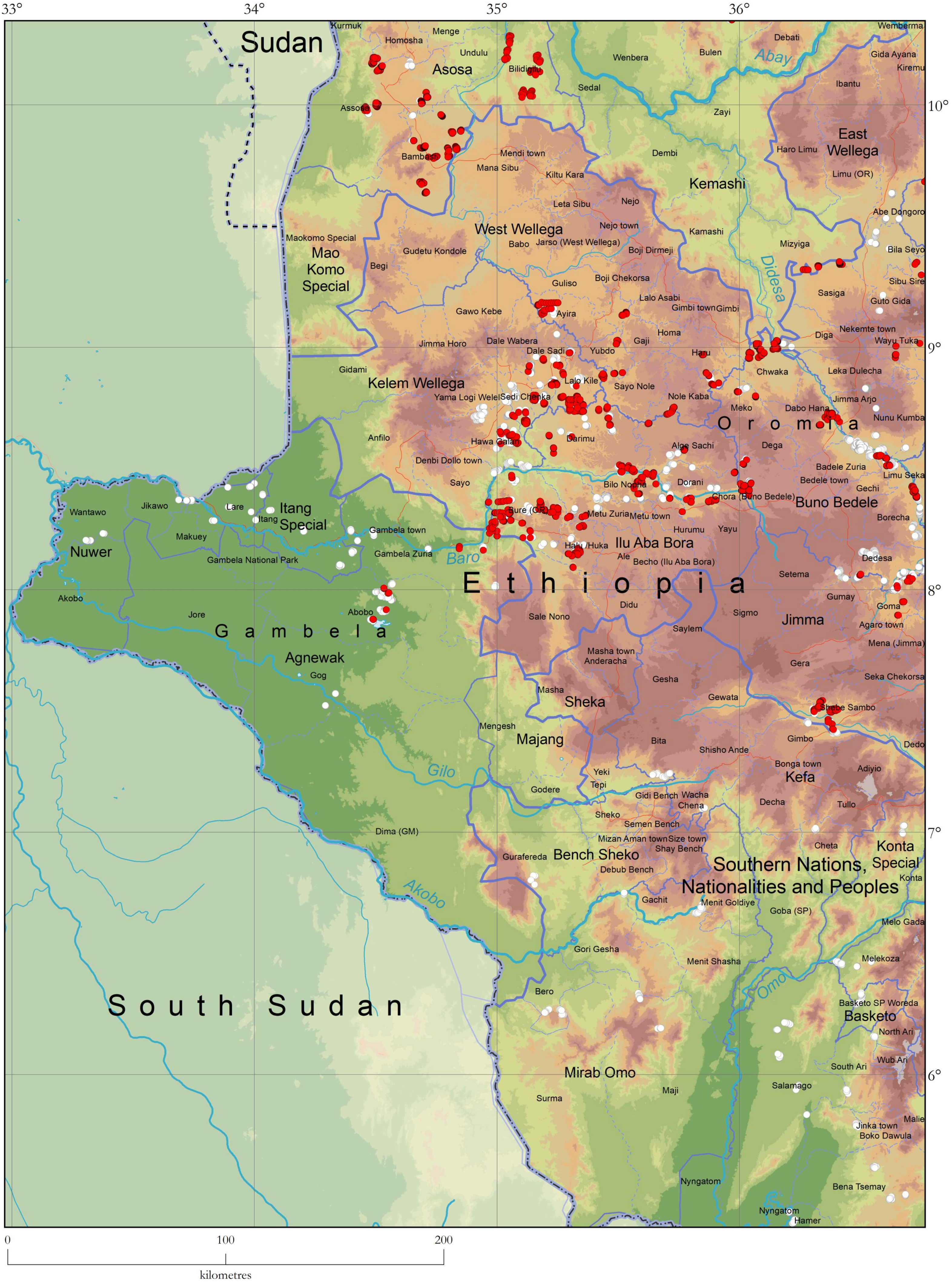

Glossina morsitans submorsitans (2010 - 2019)

The national atlas of tsetse and African animal trypanosomosis in Ethiopia

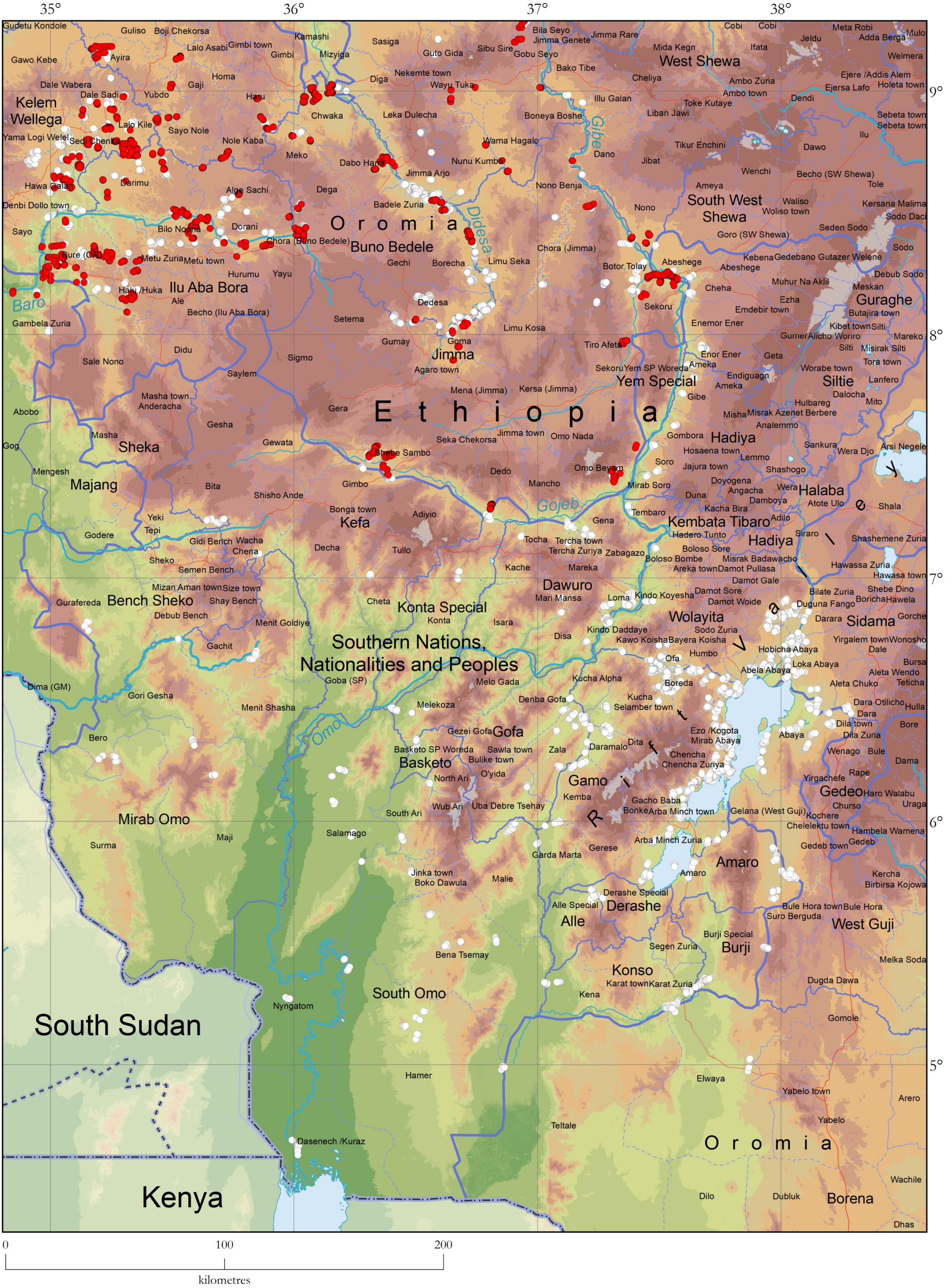

Glossina longipennis (2010 - 2019)

The national atlas of tsetse and African animal trypanosomosis in Ethiopia

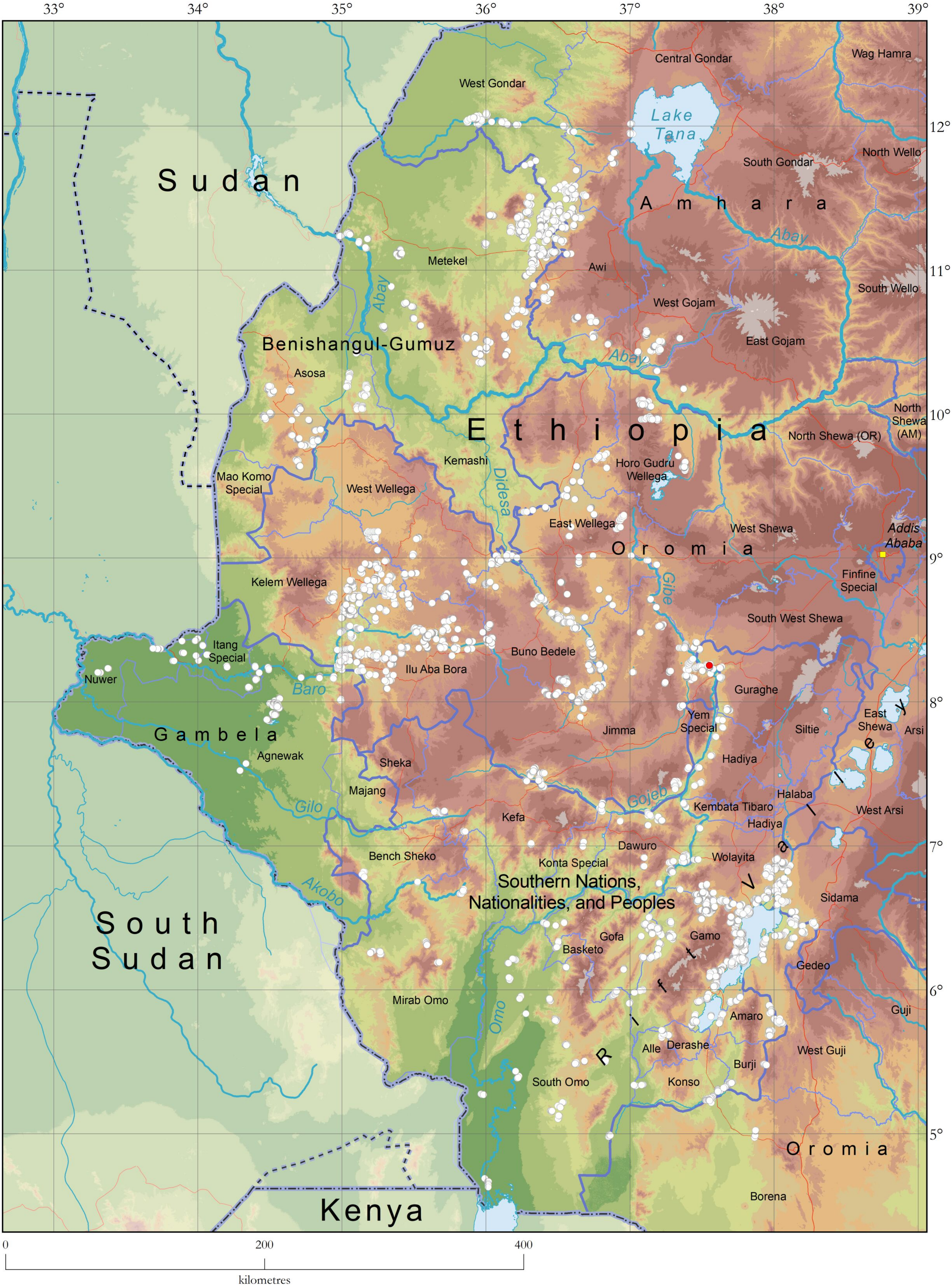

Glossina longipennis (2010 - 2019)

The national atlas of tsetse and African animal trypanosomosis in Ethiopia

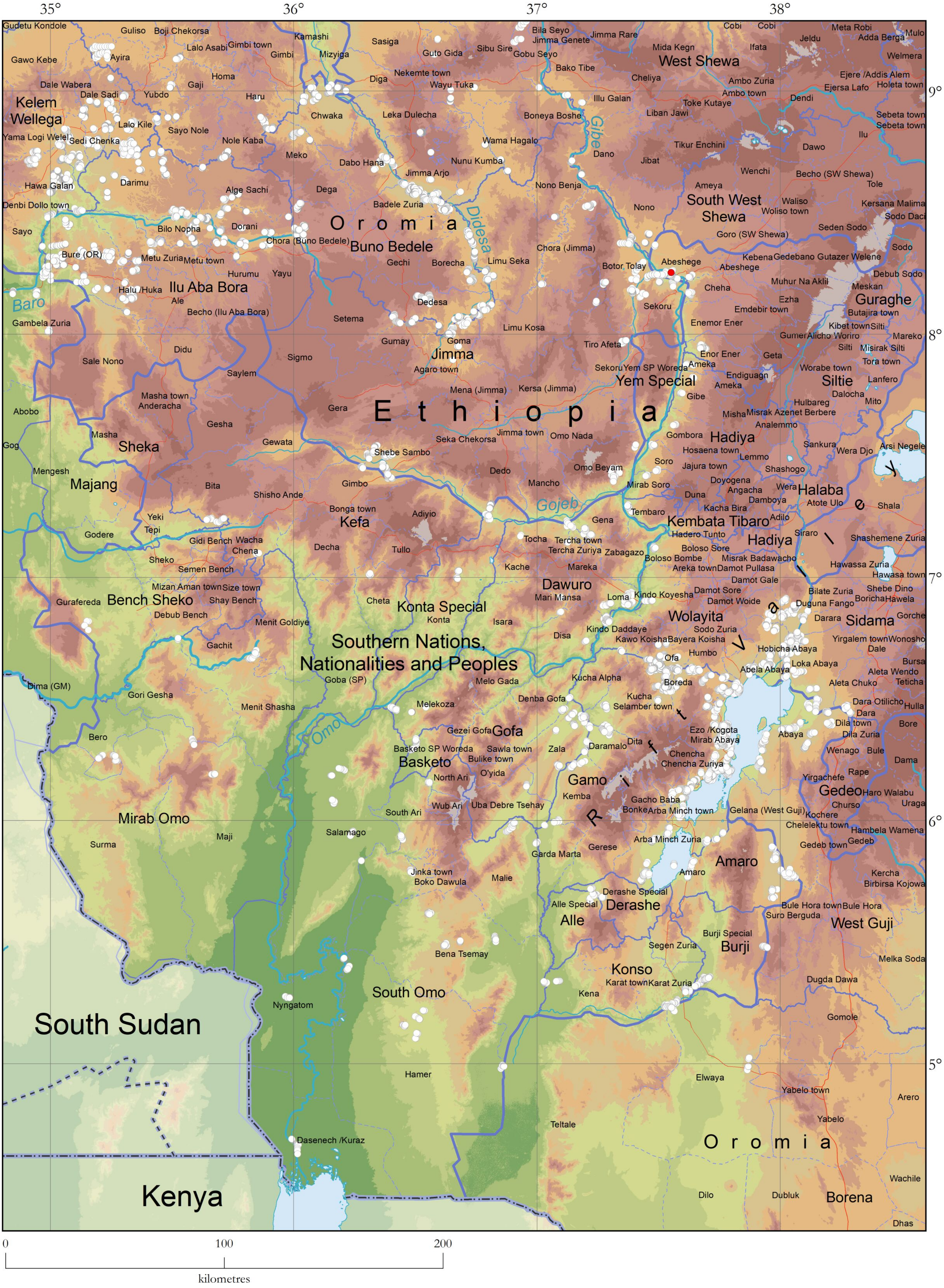

Glossina fuscipes fuscipes (2010 - 2019)

The national atlas of tsetse and African animal trypanosomosis in Ethiopia

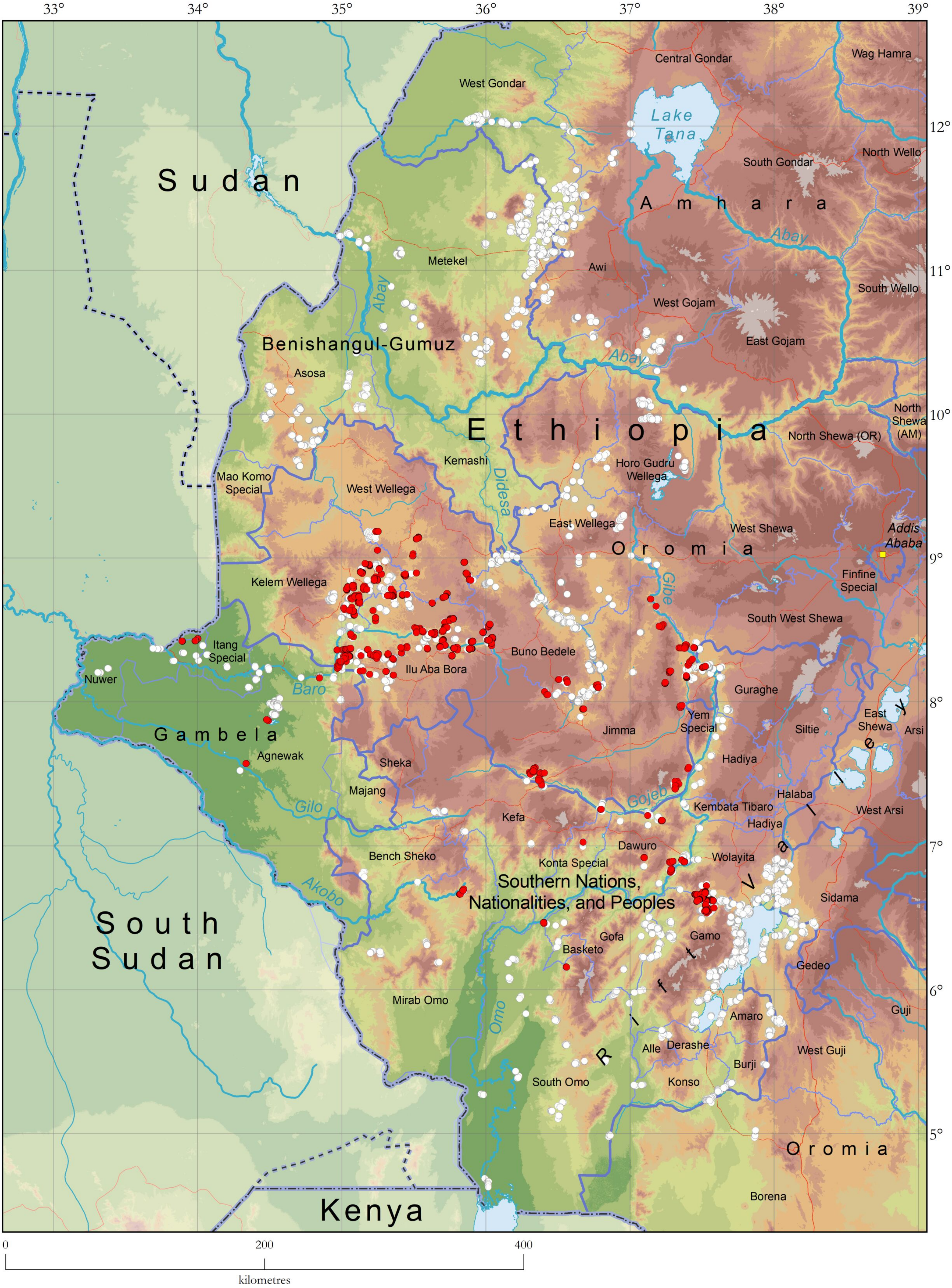

Glossina fuscipes fuscipes (2010 - 2019)

The national atlas of tsetse and African animal trypanosomosis in Ethiopia

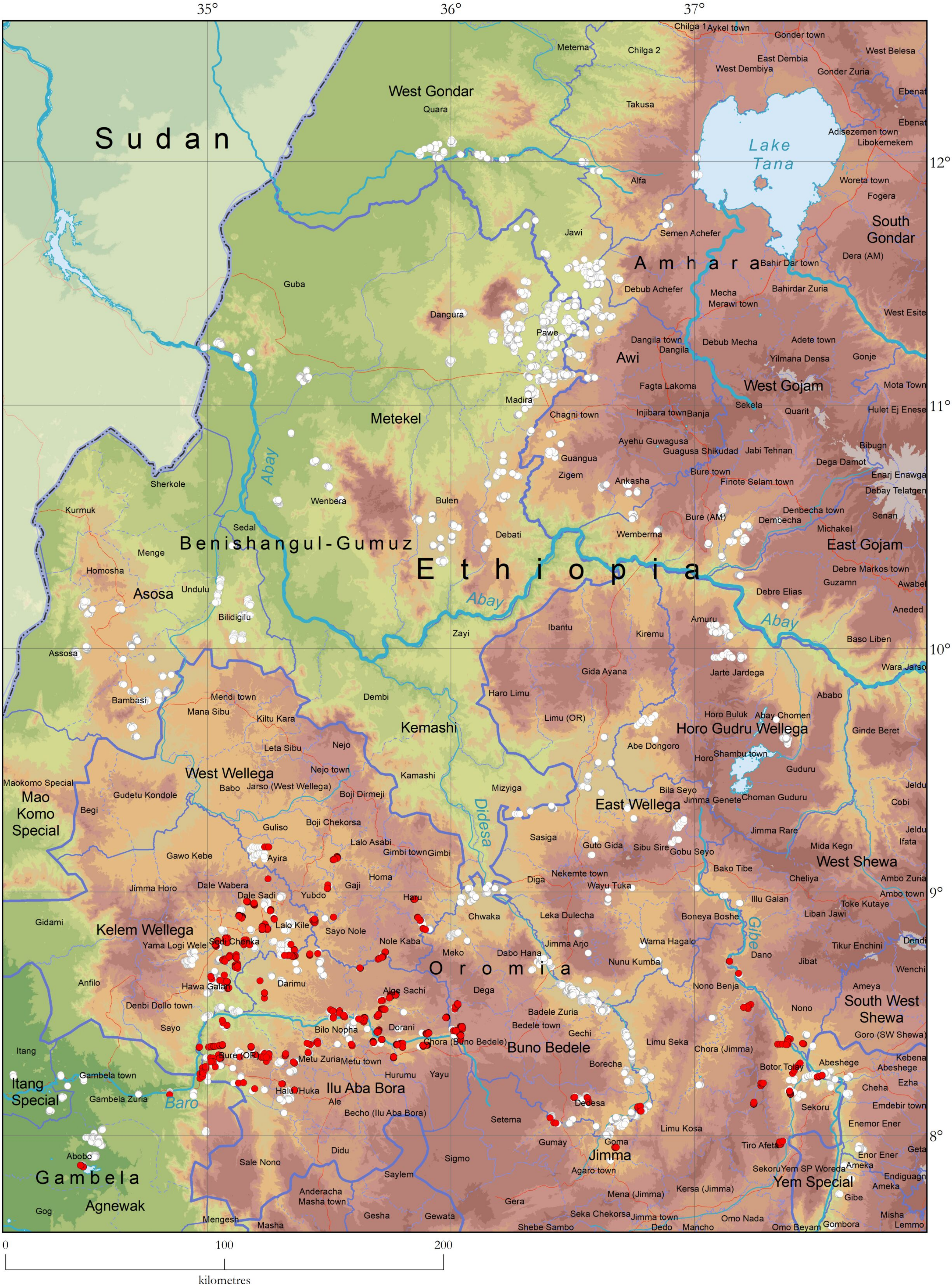

Glossina fuscipes fuscipes (2010 - 2019)

The national atlas of tsetse and African animal trypanosomosis in Ethiopia

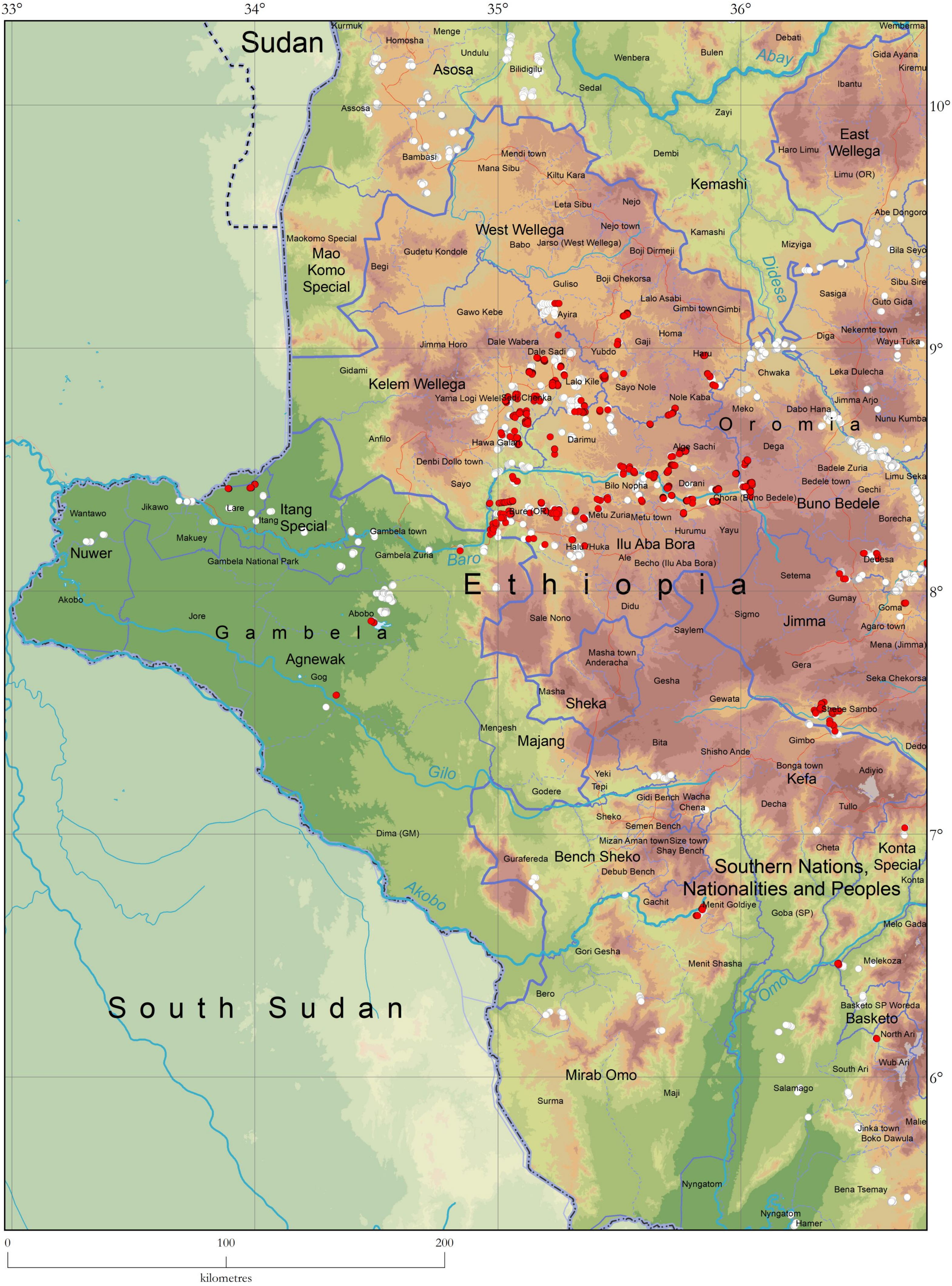

Glossina fuscipes fuscipes (2010 - 2019)

The national atlas of tsetse and African animal trypanosomosis in Ethiopia

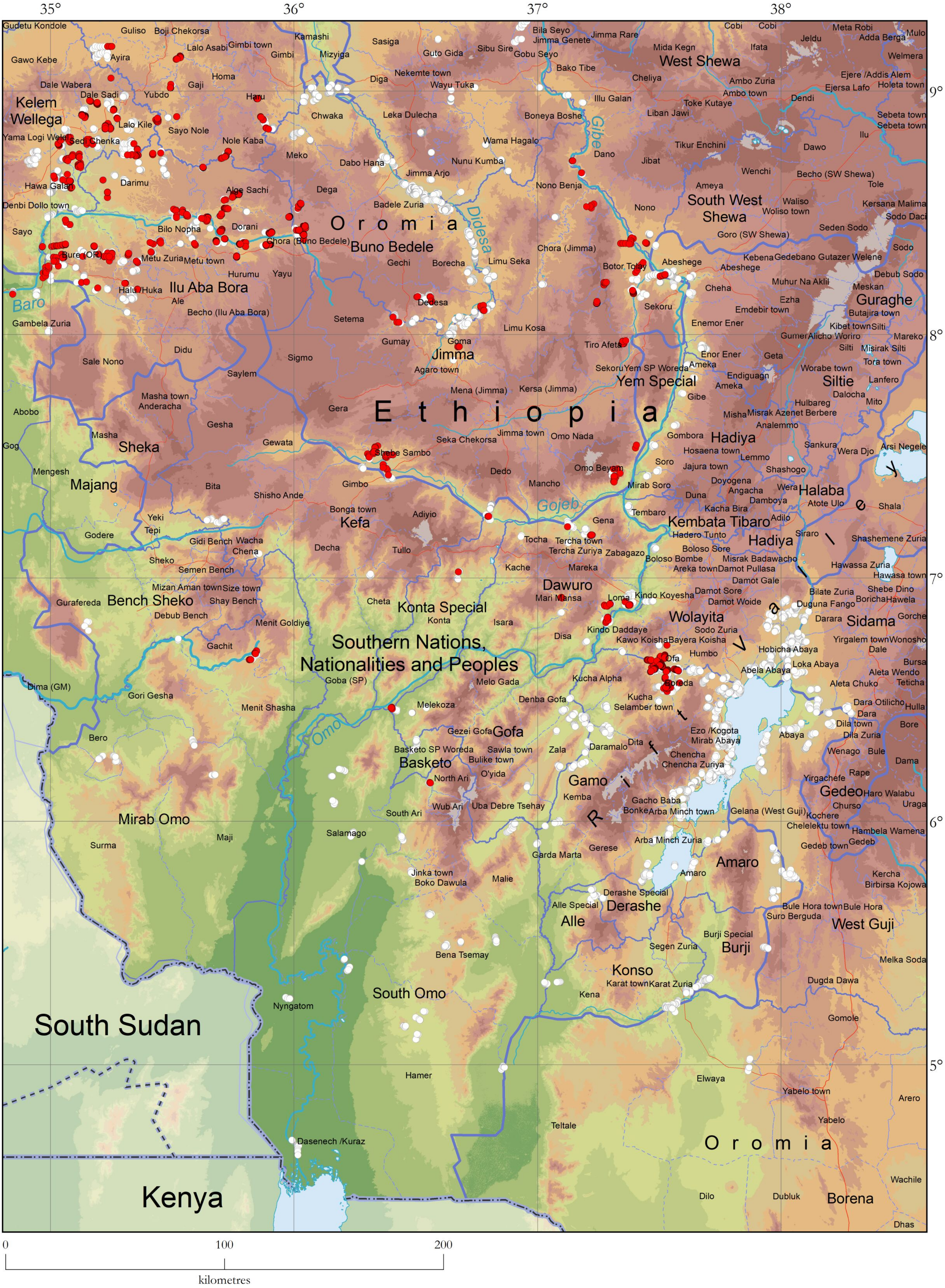

Glossina tachinoides (2010 - 2019)

The national atlas of tsetse and African animal trypanosomosis in Ethiopia

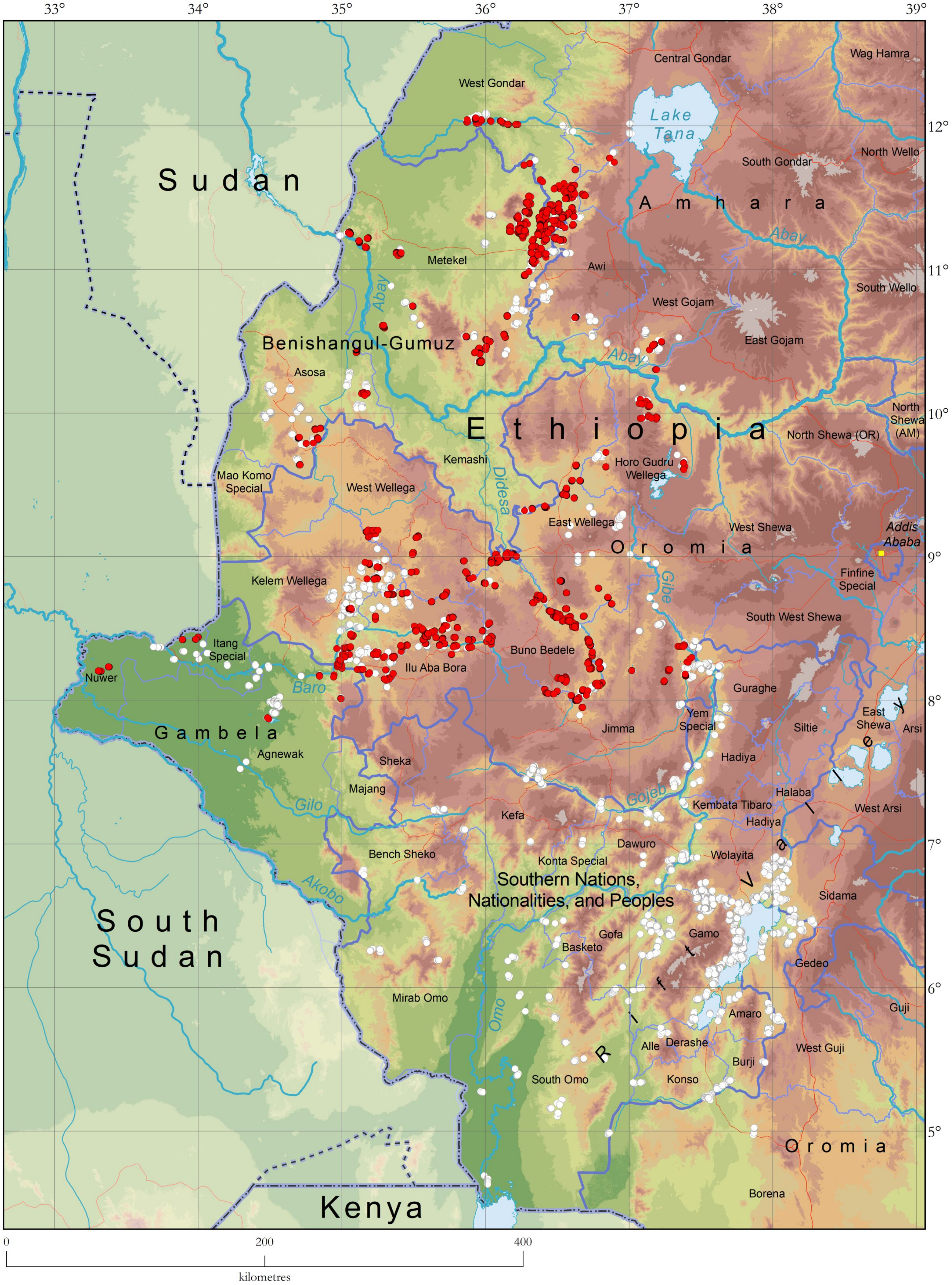

Glossina tachinoides (2010 - 2019)

The national atlas of tsetse and African animal trypanosomosis in Ethiopia

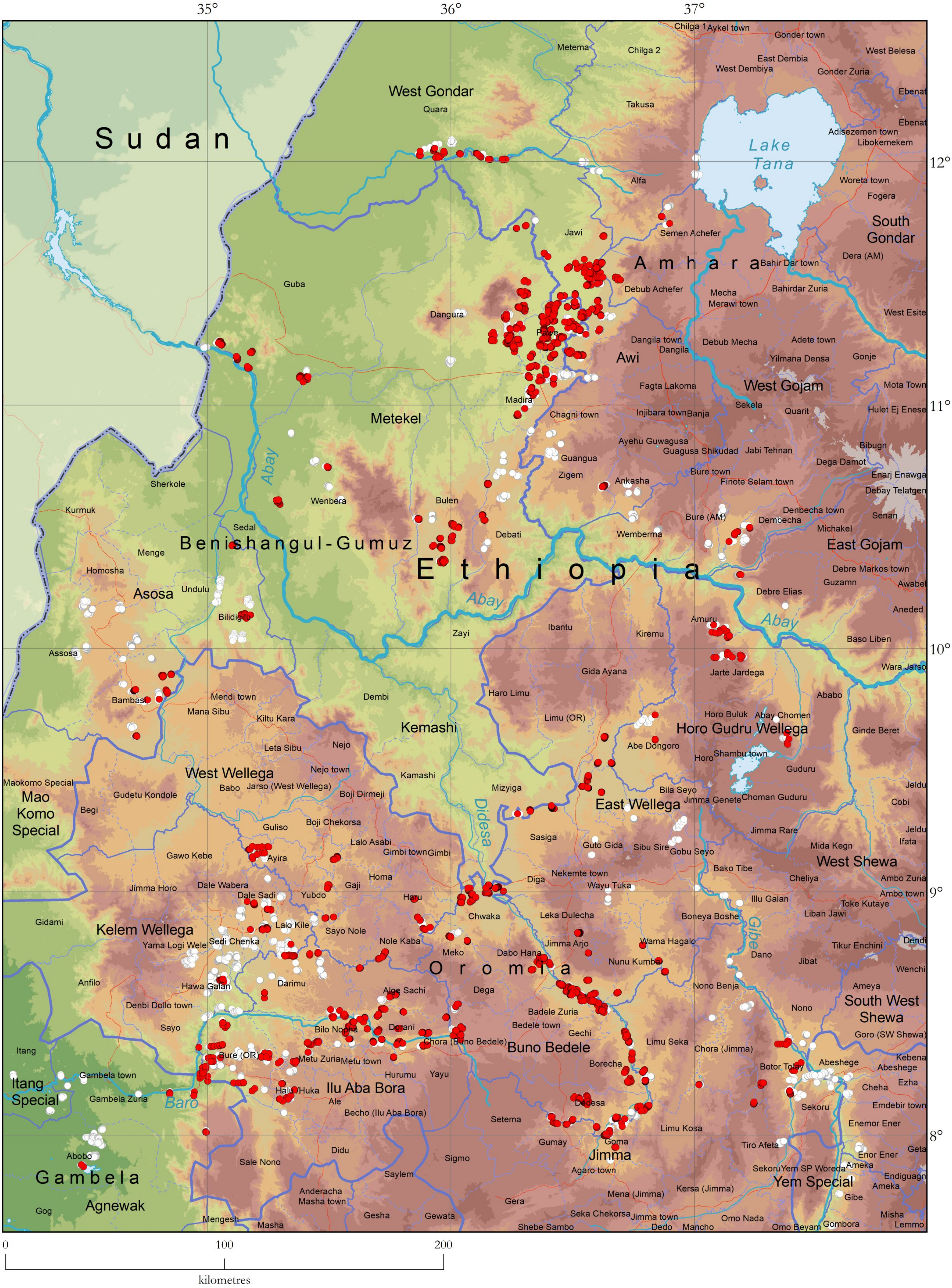

Glossina tachinoides (2010 - 2019)

The national atlas of tsetse and African animal trypanosomosis in Ethiopia

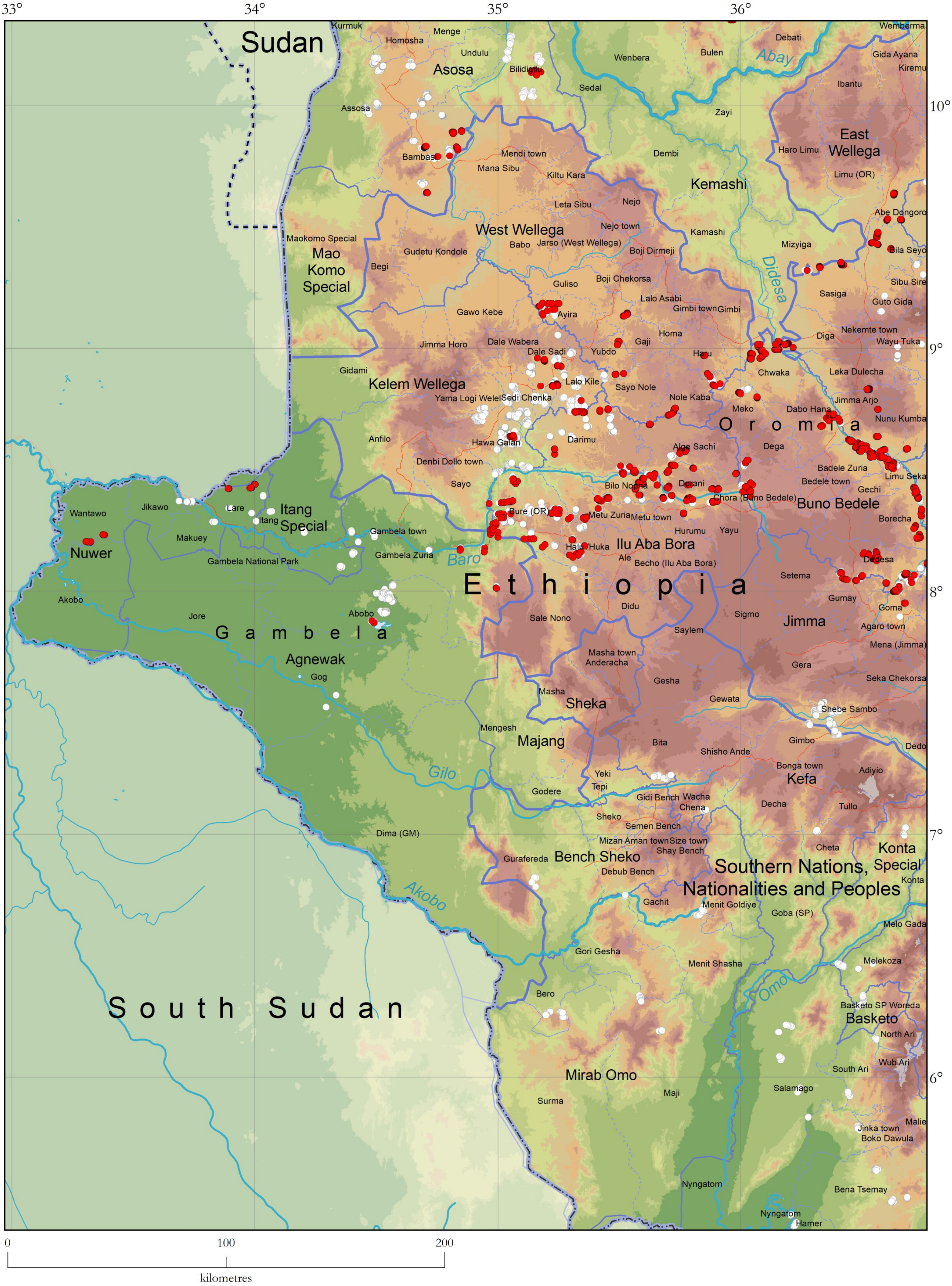

Glossina tachinoides (2010 - 2019)

The national atlas of tsetse and African animal trypanosomosis in Ethiopia

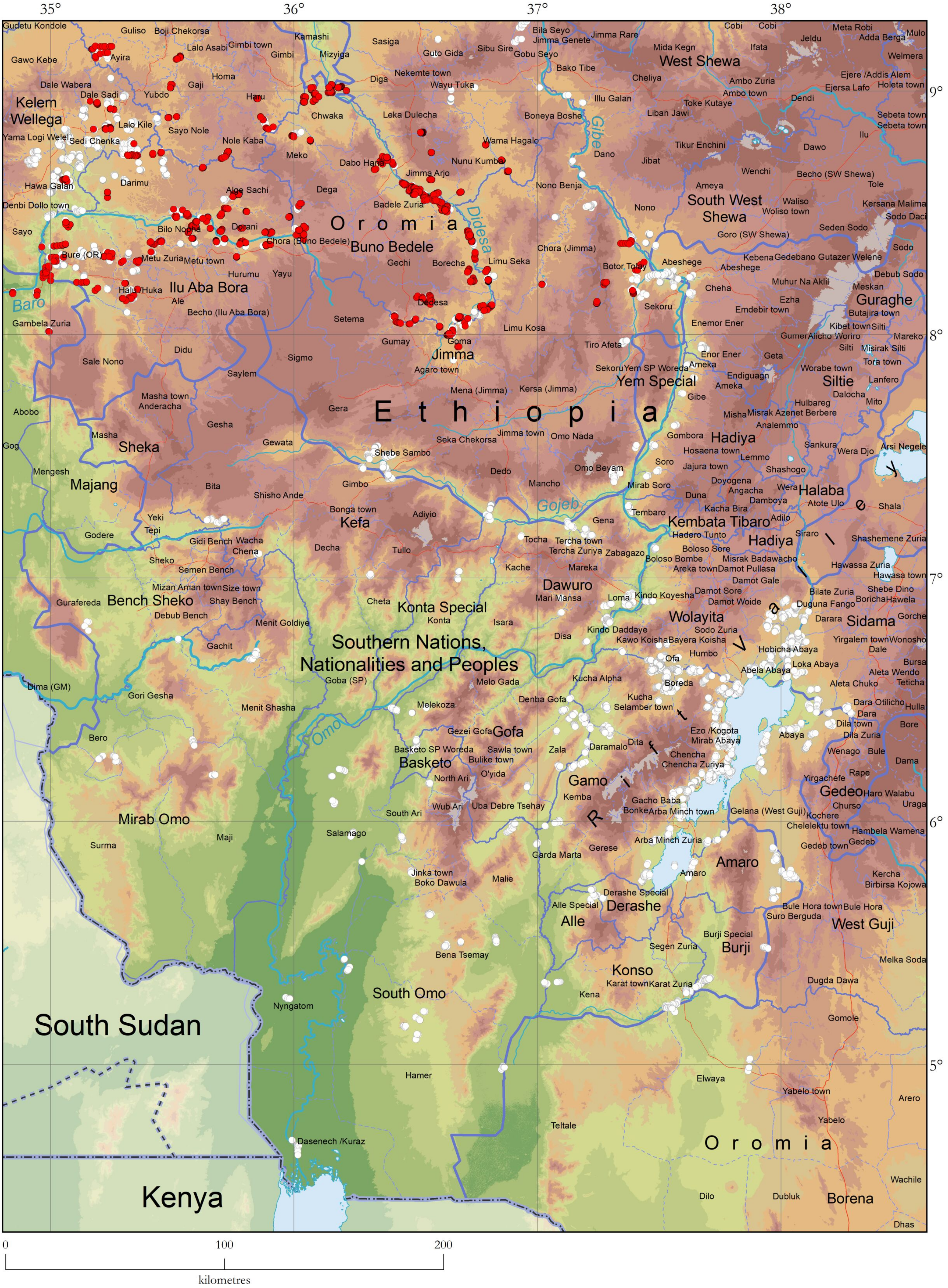

Supplement: Supplementary file 3 — Additional file 3: S3. Presence and absence (surveyed but not detected) of tsetse fly species in Ethiopia. Data collection period: 2010–2019. [file 13071_2022_5617_MOESM3_ESM.pdf]
