# Supplementary material for: The national atlas of tsetse flies and African animal trypanosomosis in Ethiopia
Source: Parasit Vectors. 2022 Dec 28;15:491. doi: 10.1186/s13071-022-05617-9 (PMC9798648; doi:10.1186/s13071-022-05617-9)
Supplement: Supplementary file 4 — Additional file 4: S4. Prevalence of bovine trypanosomosis in Ethiopia by zone. Data collection period: 2010–2019. [file 13071_2022_5617_MOESM4_ESM.docx]

**S4 Table. Prevalence of bovine trypanosomosis in Ethiopia by zone.**

Data collection period: 2010–2019.

| **Region** | **Zone** | **Animals tested**  **[n]** | ***T. vivax*** | | ***T. congolense*** | | ***T. brucei*** | | **Total** | | **Packed Cell Volume** | | |
| --- | --- | --- | --- | --- | --- | --- | --- | --- | --- | --- | --- | --- | --- |
|  |  |  | **[n]** | **[%]** | **[n]** | **[%]** | **[n]** | **[%]** | **[n]** | **[%]** | **[%]** | | |
|  |  |  |  |  |  |  |  |  |  |  | **Positive** | **Negative** | **All** |
| Amhara | Awi | 6379 | 69 | 1.08 | 140 | 2.19 | 5 | 0.08 | 213 | 3.34 | 22.15 | 26.50 | 26.35 |
| Amhara | Central Gondar | 515 | 0 | 0.00 | 0 | 0.00 | 0 | 0.00 | 0 | 0.00 |  | 26.01 | 26.01 |
| Amhara | East Gojam | 152 | 9 | 5.92 | 14 | 9.21 | 0 | 0.00 | 23 | 15.13 | 19.22 | 27.41 | 26.17 |
| Amhara | North Gonder | 778 | 4 | 0.51 | 1 | 0.13 | 0 | 0.00 | 5 | 0.64 | 27.20 | 27.44 | 27.43 |
| Amhara | West Gojam | 5282 | 84 | 1.59 | 116 | 2.20 | 9 | 0.17 | 204 | 3.86 | 23.20 | 26.34 | 26.21 |
| Amhara | West Gondar | 376 | 11 | 2.93 | 18 | 4.79 | 0 | 0.00 | 29 | 7.71 | 23.69 | 27.42 | 27.13 |
| Benshangul Gumuz | Assosa | 4706 | 73 | 1.55 | 413 | 8.78 | 2 | 0.04 | 470 | 9.99 | 22.77 | 25.42 | 25.16 |
| Benshangul Gumuz | Mao Komo | 139 | 2 | 1.44 | 14 | 10.07 | 0 | 0.00 | 15 | 10.79 | 22.13 | 22.97 | 22.88 |
| Benshangul Gumuz | Metekel | 15,045 | 194 | 1.29 | 388 | 2.58 | 16 | 0.11 | 587 | 3.90 | 23.06 | 26.61 | 26.48 |
| Gambela | Agnuwak | 728 | 4 | 0.55 | 6 | 0.82 | 0 | 0.00 | 10 | 1.37 | 24.20 | 29.03 | 28.97 |
| Gambela | Nuer | 1096 | 1 | 0.09 | 3 | 0.27 | 0 | 0.00 | 4 | 0.36 | 20.75 | 30.34 | 30.31 |
| Oromia | Buno Bedele | 5274 | 117 | 2.22 | 110 | 2.09 | 2 | 0.04 | 227 | 4.30 | 24.11 | 26.87 | 26.75 |
| Oromia | East Wellega | 5240 | 146 | 2.79 | 128 | 2.44 | 2 | 0.04 | 276 | 5.27 | 23.83 | 25.80 | 25.70 |
| Oromia | Horo Gudru | 1581 | 48 | 3.04 | 27 | 1.71 | 0 | 0.00 | 94 | 5.95 | 23.69 | 27.36 | 27.14 |
| Oromia | Ilu Aba Bora | 10,712 | 221 | 2.06 | 264 | 2.46 | 10 | 0.09 | 479 | 4.47 | 22.19 | 26.33 | 26.15 |
| Oromia | Jimma | 7543 | 163 | 2.16 | 134 | 1.78 | 0 | 0.00 | 290 | 3.84 | 21.91 | 27.12 | 26.92 |
| Oromia | Qelem Wellega | 6050 | 122 | 2.02 | 357 | 5.90 | 22 | 0.36 | 483 | 7.98 | 23.49 | 25.88 | 25.69 |
| Oromia | West Guji | 1466 | 7 | 0.48 | 76 | 5.18 | 0 | 0.00 | 82 | 5.59 | 19.60 | 23.92 | 23.68 |
| Oromia | West Shewa | 1150 | 24 | 2.09 | 41 | 3.57 | 0 | 0.00 | 65 | 5.65 | 20.40 | 27.09 | 26.71 |
| Oromia | West Wellega | 1260 | 12 | 0.95 | 16 | 1.27 | 0 | 0.00 | 28 | 2.22 | 22.64 | 26.39 | 26.31 |
| SNNP | Amaro | 265 | 7 | 2.64 | 2 | 0.75 | 0 | 0.00 | 9 | 3.40 | 23.78 | 25.46 | 25.40 |
| SNNP | Bench_Sheko | 301 | 0 | 0.00 | 1 | 0.33 | 0 | 0.00 | 1 | 0.33 | 22.00 | 27.44 | 27.42 |
| SNNP | Dawuro | 1037 | 14 | 1.35 | 30 | 2.89 | 1 | 0.10 | 44 | 4.24 | 21.42 | 23.76 | 23.66 |
| SNNP | Gamo | 3803 | 73 | 1.92 | 123 | 3.23 | 0 | 0.00 | 193 | 5.07 | 19.17 | 23.92 | 23.68 |
| SNNP | Gofa | 566 | 7 | 1.24 | 1 | 0.18 | 0 | 0.00 | 8 | 1.41 | 22.19 | 24.15 | 24.13 |
| SNNP | Guraghe | 196 | 9 | 4.59 | 7 | 3.57 | 0 | 0.00 | 16 | 8.16 | 22.63 | 21.90 | 21.96 |
| SNNP | Hadiya | 96 | 2 | 2.08 | 0 | 0.00 | 0 | 0.00 | 2 | 2.08 | 21.50 | 23.47 | 23.43 |
| SNNP | Kefa | 599 | 9 | 1.50 | 5 | 0.83 | 0 | 0.00 | 14 | 2.34 | 24.93 | 25.84 | 25.82 |
| SNNP | Kembata Timbaro | 48 | 2 | 4.17 | 2 | 4.17 | 0 | 0.00 | 4 | 8.33 | 20.50 | 22.34 | 22.19 |
| SNNP | Konso | 396 | 2 | 0.51 | 7 | 1.77 | 0 | 0.00 | 8 | 2.02 | 24.25 | 25.92 | 25.89 |
| SNNP | Sidama | 1714 | 24 | 1.40 | 77 | 4.49 | 2 | 0.12 | 96 | 5.60 | 19.09 | 23.40 | 23.16 |
| SNNP | South Omo | 593 | 3 | 0.51 | 8 | 1.35 | 0 | 0.00 | 11 | 1.85 | 20.09 | 23.74 | 23.67 |
| SNNP | West Omo | 562 | 33 | 5.87 | 5 | 0.89 | 0 | 0.00 | 37 | 6.58 | 22.54 | 25.42 | 25.23 |
| SNNP | Wolayta | 2133 | 60 | 2.81 | 147 | 6.89 | 2 | 0.09 | 207 | 9.70 | 20.55 | 22.82 | 22.60 |
| SNNP | Yem Special woreda | 222 | 3 | 1.35 | 4 | 1.80 | 1 | 0.45 | 7 | 3.15 | 24.71 | 26.27 | 26.23 |
| TOTAL |  | 88,003 | 1559 | 1.77 | 2685 | 3.05 | 74 | 0.08 | 4241 | 4.82 | 22.44 | 26.13 | 25.95 |
