# Supplementary material for: The national atlas of tsetse flies and African animal trypanosomosis in Ethiopia
Source: Parasit Vectors. 2022 Dec 28;15:491. doi: 10.1186/s13071-022-05617-9 (PMC9798648; doi:10.1186/s13071-022-05617-9)
Supplement: Supplementary file 5 — Additional file 5: S5. Prevalence of bovine trypanosomosis in Ethiopia by district (woreda). Data collection period: 2010–2019. [file 13071_2022_5617_MOESM5_ESM.docx]

**S5 Table. Prevalence of bovine trypanosomosis in Ethiopia by district (*woreda*).**

Data collection period: 2010–2019.

| **Region** | **Zone** | **Woreda** | **Animals tested**  **[n]** | ***T. vivax*** | | ***T. congolense*** | | ***T. brucei*** | | **Total** | | **Packed Cell Volume** | | |
| --- | --- | --- | --- | --- | --- | --- | --- | --- | --- | --- | --- | --- | --- | --- |
|  |  |  |  | **[n]** | **[%]** | **[n]** | **[%]** | **[n]** | **[%]** | **[n]** | **[%]** | **[%]** | | |
|  |  |  |  |  |  |  |  |  |  |  |  | **Positive** | **Negative** | **All** |
| Amhara | Awi | Ankasha Guwagussa | 1460 | 1 | 0.07 | 9 | 0.62 | 0 | 0.00 | 10 | 0.68 | 23.00 | 27.46 | 27.43 |
| Amhara | Awi | Guangua | 533 | 1 | 0.19 | 0 | 0.00 | 0 | 0.00 | 1 | 0.19 | 30.00 | 25.03 | 25.04 |
| Amhara | Awi | Jawi | 4386 | 67 | 1.53 | 131 | 2.99 | 5 | 0.11 | 202 | 4.61 | 22.06 | 26.35 | 26.15 |
| Amhara | Central Gondar | Alfa | 515 | 0 | 0.00 | 0 | 0.00 | 0 | 0.00 | 0 | 0.00 |  | 26.01 | 26.01 |
| Amhara | Central Gondar | Takusa | 778 | 4 | 0.51 | 1 | 0.13 | 0 | 0.00 | 5 | 0.64 | 27.20 | 27.44 | 27.43 |
| Amhara | East Gojam | Debre Elias | 152 | 9 | 5.92 | 14 | 9.21 | 0 | 0.00 | 23 | 15.13 | 19.22 | 27.41 | 26.17 |
| Amhara | West Gojam | Bure (AM) | 976 | 0 | 0.00 | 28 | 2.87 | 1 | 0.10 | 28 | 2.87 | 25.21 | 27.07 | 27.02 |
| Amhara | West Gojam | Dembecha | 271 | 10 | 3.69 | 22 | 8.12 | 0 | 0.00 | 32 | 11.81 | 22.00 | 28.03 | 27.32 |
| Amhara | West Gojam | Jabitehinan | 51 | 0 | 0.00 | 0 | 0.00 | 0 | 0.00 | 0 | 0.00 |  | 27.61 | 27.61 |
| Amhara | West Gojam | North Achefer | 1024 | 13 | 1.27 | 11 | 1.07 | 0 | 0.00 | 24 | 2.34 | 23.50 | 27.46 | 27.37 |
| Amhara | West Gojam | South Achefer | 2542 | 60 | 2.36 | 46 | 1.81 | 8 | 0.31 | 110 | 4.33 | 22.35 | 25.16 | 25.04 |
| Amhara | West Gojam | Wenberma | 418 | 1 | 0.24 | 9 | 2.15 | 0 | 0.00 | 10 | 2.39 | 29.90 | 27.71 | 27.76 |
| Amhara | West Gondar | Quara | 376 | 11 | 2.93 | 18 | 4.79 | 0 | 0.00 | 29 | 7.71 | 23.69 | 27.42 | 27.13 |
| Benshangul Gumuz | Assosa | Assosa | 2904 | 32 | 1.10 | 262 | 9.02 | 0 | 0.00 | 289 | 9.95 | 22.38 | 24.86 | 24.62 |
| Benshangul Gumuz | Assosa | Bambasi | 1802 | 41 | 2.28 | 151 | 8.38 | 2 | 0.11 | 181 | 10.04 | 23.38 | 26.32 | 26.03 |
| Benshangul Gumuz | Mao Komo | Mao Komo | 139 | 2 | 1.44 | 14 | 10.07 | 0 | 0.00 | 15 | 10.79 | 22.13 | 22.97 | 22.88 |
| Benshangul Gumuz | Metekel | Bulen | 2103 | 18 | 0.86 | 20 | 0.95 | 0 | 0.00 | 32 | 1.52 | 23.97 | 28.01 | 27.95 |
| Benshangul Gumuz | Metekel | Dangura | 2386 | 46 | 1.93 | 119 | 4.99 | 1 | 0.04 | 163 | 6.83 | 23.90 | 26.73 | 26.53 |
| Benshangul Gumuz | Metekel | Dibate | 2331 | 46 | 1.97 | 25 | 1.07 | 7 | 0.30 | 78 | 3.35 | 23.53 | 26.30 | 26.21 |
| Benshangul Gumuz | Metekel | Guba | 301 | 0 | 0.00 | 0 | 0.00 | 0 | 0.00 | 0 | 0.00 |  | 26.30 | 26.30 |
| Benshangul Gumuz | Metekel | Mandura | 2597 | 12 | 0.46 | 12 | 0.46 | 0 | 0.00 | 22 | 0.85 | 24.41 | 26.59 | 26.57 |
| Benshangul Gumuz | Metekel | Pawe | 4946 | 68 | 1.37 | 201 | 4.06 | 8 | 0.16 | 277 | 5.60 | 22.60 | 26.23 | 26.03 |
| Benshangul Gumuz | Metekel | Wembera | 381 | 4 | 1.05 | 11 | 2.89 | 0 | 0.00 | 15 | 3.94 | 16.13 | 25.30 | 24.94 |
| Gambela | Agnuwak | Gambela zuria | 528 | 4 | 0.76 | 6 | 1.14 | 0 | 0.00 | 10 | 1.89 | 24.20 | 29.08 | 28.98 |
| Gambela | Agnuwak | Jore | 200 | 0 | 0.00 | 0 | 0.00 | 0 | 0.00 | 0 | 0.00 |  | 28.92 | 28.92 |
| Gambela | Nuer | Jikawo | 368 | 0 | 0.00 | 0 | 0.00 | 0 | 0.00 | 0 | 0.00 |  | 30.12 | 30.12 |
| Gambela | Nuer | Lare | 591 | 1 | 0.17 | 3 | 0.51 | 0 | 0.00 | 4 | 0.68 | 20.75 | 30.03 | 29.97 |
| Gambela | Nuer | Makuey | 137 | 0 | 0.00 | 0 | 0.00 | 0 | 0.00 | 0 | 0.00 |  | 32.26 | 32.26 |
| Oromia | Buno Bedele | Bedele Zuriya | 1255 | 16 | 1.27 | 32 | 2.55 | 0 | 0.00 | 46 | 3.67 | 26.70 | 27.85 | 27.80 |
| Oromia | Buno Bedele | Boricha | 326 | 17 | 5.21 | 7 | 2.15 | 0 | 0.00 | 24 | 7.36 | 24.13 | 25.97 | 25.83 |
| Oromia | Buno Bedele | Chawaqa | 864 | 14 | 1.62 | 10 | 1.16 | 0 | 0.00 | 24 | 2.78 | 23.67 | 25.99 | 25.92 |
| Oromia | Buno Bedele | Chora | 868 | 12 | 1.38 | 22 | 2.53 | 0 | 0.00 | 34 | 3.92 | 25.24 | 28.68 | 28.54 |
| Oromia | Buno Bedele | Dabo Hana | 401 | 8 | 2.00 | 10 | 2.49 | 0 | 0.00 | 18 | 4.49 | 25.06 | 26.46 | 26.40 |
| Oromia | Buno Bedele | Didessa | 600 | 9 | 1.50 | 13 | 2.17 | 0 | 0.00 | 22 | 3.67 | 20.73 | 26.12 | 25.92 |
| Oromia | Buno Bedele | Gechi | 948 | 43 | 4.54 | 22 | 2.32 | 2 | 0.21 | 67 | 7.07 | 23.64 | 25.96 | 25.80 |
| Oromia | Buno Bedele | Meko | 413 | 6 | 1.45 | 4 | 0.97 | 0 | 0.00 | 10 | 2.42 | 20.00 | 25.98 | 25.84 |
| Oromia | East Wellega | Bila Seyo | 385 | 4 | 1.04 | 4 | 1.04 | 0 | 0.00 | 8 | 2.08 | 24.00 | 26.81 | 26.76 |
| Oromia | East Wellega | Bilo Boshe (Boneya_Boshe) | 811 | 22 | 2.71 | 32 | 3.95 | 0 | 0.00 | 54 | 6.66 | 23.44 | 25.50 | 25.36 |
| Oromia | East Wellega | Diga | 584 | 18 | 3.08 | 6 | 1.03 | 1 | 0.17 | 25 | 4.28 | 25.52 | 25.84 | 25.82 |
| Oromia | East Wellega | Gida Ayana | 473 | 15 | 3.17 | 18 | 3.81 | 0 | 0.00 | 33 | 6.98 | 21.18 | 25.39 | 25.09 |
| Oromia | East Wellega | Jimma Arjo | 414 | 12 | 2.90 | 6 | 1.45 | 0 | 0.00 | 18 | 4.35 | 25.22 | 26.28 | 26.23 |
| Oromia | East Wellega | Nunu Kumba | 601 | 19 | 3.16 | 8 | 1.33 | 1 | 0.17 | 28 | 4.66 | 25.86 | 26.12 | 26.10 |
| Oromia | East Wellega | Sibu Sire | 648 | 3 | 0.46 | 7 | 1.08 | 0 | 0.00 | 10 | 1.54 | 24.30 | 25.53 | 25.51 |
| Oromia | East Wellega | Wama Hagelo | 601 | 16 | 2.66 | 4 | 0.67 | 0 | 0.00 | 20 | 3.33 | 24.60 | 25.92 | 25.88 |
| Oromia | East Wellega | Wayu Tuqa | 723 | 37 | 5.12 | 43 | 5.95 | 0 | 0.00 | 80 | 11.07 | 23.36 | 25.41 | 25.18 |
| Oromia | Horo Gudru | Abay chomen | 472 | 16 | 3.39 | 10 | 2.12 | 0 | 0.00 | 25 | 5.30 | 25.28 | 27.54 | 27.42 |
| Oromia | Horo Gudru | Abe Dengoro | 343 | 17 | 4.96 | 10 | 2.92 | 0 | 0.00 | 27 | 7.87 | 23.11 | 25.53 | 25.34 |
| Oromia | Horo Gudru | Amuru | 383 | 15 | 3.92 | 7 | 1.83 | 0 | 0.00 | 22 | 5.74 | 23.27 | 28.04 | 27.77 |
| Oromia | Horo Gudru | Jarte Jardega | 383 | 0 | 0.00 | 0 | 0.00 | 0 | 0.00 | 20 | 5.22 | 22.95 | 28.03 | 27.77 |
| Oromia | Ilu Aba Bora | Algesachi | 576 | 7 | 1.22 | 17 | 2.95 | 0 | 0.00 | 24 | 4.17 | 24.42 | 27.52 | 27.39 |
| Oromia | Ilu Aba Bora | Bilo Nopha | 936 | 22 | 2.35 | 10 | 1.07 | 1 | 0.11 | 31 | 3.31 | 19.87 | 26.57 | 26.35 |
| Oromia | Ilu Aba Bora | Bure (OR) | 2146 | 41 | 1.91 | 69 | 3.22 | 2 | 0.09 | 110 | 5.13 | 22.40 | 25.71 | 25.54 |
| Oromia | Ilu Aba Bora | Darimu | 3037 | 79 | 2.60 | 95 | 3.13 | 4 | 0.13 | 170 | 5.60 | 22.23 | 25.67 | 25.48 |
| Oromia | Ilu Aba Bora | Doreni | 945 | 30 | 3.17 | 30 | 3.17 | 0 | 0.00 | 58 | 6.14 | 21.98 | 26.13 | 25.88 |
| Oromia | Ilu Aba Bora | Halu/Huka | 864 | 10 | 1.16 | 8 | 0.93 | 0 | 0.00 | 18 | 2.08 | 19.44 | 25.63 | 25.50 |
| Oromia | Ilu Aba Bora | Hurumu | 415 | 6 | 1.45 | 2 | 0.48 | 1 | 0.24 | 9 | 2.17 | 18.78 | 27.88 | 27.68 |
| Oromia | Ilu Aba Bora | Metu | 912 | 9 | 0.99 | 19 | 2.08 | 0 | 0.00 | 26 | 2.85 | 23.23 | 28.65 | 28.50 |
| Oromia | Ilu Aba Bora | Yayo | 480 | 9 | 1.88 | 4 | 0.83 | 2 | 0.42 | 15 | 3.13 | 22.47 | 27.07 | 26.93 |
| Oromia | Jimma | Boter Tolay | 334 | 36 | 10.78 | 4 | 1.20 | 0 | 0.00 | 40 | 11.98 | 19.85 | 26.72 | 25.90 |
| Oromia | Jimma | Dedo | 648 | 11 | 1.70 | 11 | 1.70 | 0 | 0.00 | 22 | 3.40 | 25.86 | 28.76 | 28.66 |
| Oromia | Jimma | Gera | 102 | 0 | 0.00 | 0 | 0.00 | 0 | 0.00 | 0 | 0.00 |  | 29.28 | 29.28 |
| Oromia | Jimma | Gomma | 1459 | 13 | 0.89 | 7 | 0.48 | 0 | 0.00 | 20 | 1.37 | 22.50 | 27.42 | 27.35 |
| Oromia | Jimma | Gumay | 1152 | 12 | 1.04 | 6 | 0.52 | 0 | 0.00 | 18 | 1.56 | 24.00 | 27.55 | 27.49 |
| Oromia | Jimma | Limu kosa | 392 | 4 | 1.02 | 15 | 3.83 | 0 | 0.00 | 19 | 4.85 | 22.26 | 28.41 | 28.11 |
| Oromia | Jimma | Limu Seka | 410 | 29 | 7.07 | 6 | 1.46 | 0 | 0.00 | 35 | 8.54 | 22.37 | 27.21 | 26.80 |
| Oromia | Jimma | Omo Beyam | 388 | 14 | 3.61 | 18 | 4.64 | 0 | 0.00 | 27 | 6.96 | 21.00 | 25.70 | 25.37 |
| Oromia | Jimma | Omo Nada | 150 | 0 | 0.00 | 0 | 0.00 | 0 | 0.00 | 0 | 0.00 |  | 25.42 | 25.42 |
| Oromia | Jimma | Sekoru | 546 | 10 | 1.83 | 24 | 4.40 | 0 | 0.00 | 32 | 5.86 | 23.16 | 26.34 | 26.15 |
| Oromia | Jimma | Shebe Sombo | 1006 | 10 | 0.99 | 35 | 3.48 | 0 | 0.00 | 45 | 4.47 | 22.49 | 27.17 | 26.96 |
| Oromia | Jimma | Tiro Afeta | 956 | 24 | 2.51 | 8 | 0.84 | 0 | 0.00 | 32 | 3.35 | 18.22 | 25.55 | 25.30 |
| Oromia | Qelem Wellega | Dale sadi | 2627 | 45 | 1.71 | 114 | 4.34 | 9 | 0.34 | 161 | 6.13 | 24.16 | 25.72 | 25.62 |
| Oromia | Qelem Wellega | Dale Wabera/Sedichenka | 1086 | 40 | 3.68 | 152 | 14.00 | 11 | 1.01 | 195 | 17.96 | 22.76 | 25.77 | 25.23 |
| Oromia | Qelem Wellega | Hawa Gelan | 1033 | 21 | 2.03 | 53 | 5.13 | 2 | 0.19 | 75 | 7.26 | 24.63 | 25.47 | 25.41 |
| Oromia | Qelem Wellega | Laloqile | 1304 | 16 | 1.23 | 38 | 2.91 | 0 | 0.00 | 52 | 3.99 | 22.50 | 26.57 | 26.40 |
| Oromia | West Guji | Abaya | 1065 | 5 | 0.47 | 46 | 4.32 | 0 | 0.00 | 50 | 4.69 | 20.26 | 24.97 | 24.75 |
| Oromia | West Guji | Gelana | 267 | 0 | 0.00 | 20 | 7.49 | 0 | 0.00 | 20 | 7.49 | 17.40 | 20.40 | 20.18 |
| Oromia | West Guji | Suro Barguda | 134 | 2 | 1.49 | 10 | 7.46 | 0 | 0.00 | 12 | 8.96 | 20.50 | 22.27 | 22.11 |
| Oromia | West Shewa | Bako_Tibe | 402 | 5 | 1.24 | 1 | 0.25 | 0 | 0.00 | 6 | 1.49 | 20.17 | 26.81 | 26.71 |
| Oromia | West Shewa | Dano | 236 | 10 | 4.24 | 14 | 5.93 | 0 | 0.00 | 24 | 10.17 | 18.83 | 26.15 | 25.41 |
| Oromia | West Shewa | Nono | 512 | 9 | 1.76 | 26 | 5.08 | 0 | 0.00 | 35 | 6.84 | 21.51 | 27.73 | 27.31 |
| Oromia | West Wellega | Ayira | 360 | 5 | 1.39 | 5 | 1.39 | 0 | 0.00 | 10 | 2.78 | 22.30 | 25.42 | 25.33 |
| Oromia | West Wellega | Guliso | 300 | 2 | 0.67 | 2 | 0.67 | 0 | 0.00 | 4 | 1.33 | 23.25 | 27.08 | 27.03 |
| Oromia | West Wellega | Yubdo | 600 | 5 | 0.83 | 9 | 1.50 | 0 | 0.00 | 14 | 2.33 | 22.71 | 26.62 | 26.53 |
| SNNP | Amaro | Amaro | 265 | 7 | 2.64 | 2 | 0.75 | 0 | 0.00 | 9 | 3.40 | 23.78 | 25.46 | 25.40 |
| SNNP | Bench_Sheko | Semen Bench | 161 | 0 | 0.00 | 1 | 0.62 | 0 | 0.00 | 1 | 0.62 | 22.00 | 26.71 | 26.68 |
| SNNP | Bench_Sheko | Sheko | 140 | 0 | 0.00 | 0 | 0.00 | 0 | 0.00 | 0 | 0.00 |  | 28.28 | 28.28 |
| SNNP | Dawuro | Disa | 213 | 2 | 0.94 | 4 | 1.88 | 0 | 0.00 | 6 | 2.82 | 24.17 | 24.85 | 24.83 |
| SNNP | Dawuro | Gena Bossa | 100 | 2 | 2.00 | 8 | 8.00 | 0 | 0.00 | 10 | 10.00 | 18.05 | 18.72 | 18.66 |
| SNNP | Dawuro | Loma | 580 | 8 | 1.38 | 8 | 1.38 | 1 | 0.17 | 16 | 2.76 | 21.50 | 24.11 | 24.04 |
| SNNP | Dawuro | Tercha Zuria | 144 | 2 | 1.39 | 10 | 6.94 | 0 | 0.00 | 12 | 8.33 | 22.75 | 23.96 | 23.86 |
| SNNP | Gamo | Arba Minch Zuria | 1466 | 28 | 1.91 | 23 | 1.57 | 0 | 0.00 | 51 | 3.48 | 21.49 | 24.56 | 24.45 |
| SNNP | Gamo | Boreda | 138 | 0 | 0.00 | 0 | 0.00 | 0 | 0.00 | 0 | 0.00 |  | 24.64 | 24.64 |
| SNNP | Gamo | Daramalo | 266 | 0 | 0.00 | 11 | 4.14 | 0 | 0.00 | 11 | 4.14 | 20.18 | 26.06 | 25.82 |
| SNNP | Gamo | Kemba | 507 | 6 | 1.18 | 2 | 0.39 | 0 | 0.00 | 8 | 1.58 | 23.25 | 25.64 | 25.60 |
| SNNP | Gamo | Kucha | 108 | 5 | 4.63 | 1 | 0.93 | 0 | 0.00 | 6 | 5.56 | 20.17 | 23.78 | 23.58 |
| SNNP | Gamo | Mirab Abaya | 1318 | 34 | 2.58 | 86 | 6.53 | 0 | 0.00 | 117 | 8.88 | 17.73 | 21.92 | 21.55 |
| SNNP | Gofa | Denba Gofa | 159 | 0 | 0.00 | 0 | 0.00 | 0 | 0.00 | 0 | 0.00 |  | 23.87 | 23.87 |
| SNNP | Gofa | Ouba Debretsehaye | 74 | 0 | 0.00 | 0 | 0.00 | 0 | 0.00 | 0 | 0.00 |  | 27.27 | 27.27 |
| SNNP | Gofa | Zala | 333 | 7 | 2.10 | 1 | 0.30 | 0 | 0.00 | 8 | 2.40 | 22.19 | 23.58 | 23.55 |
| SNNP | Guraghe | Abeshege | 100 | 6 | 6.00 | 7 | 7.00 | 0 | 0.00 | 13 | 13.00 | 21.62 | 23.77 | 23.49 |
| SNNP | Guraghe | Cheha | 96 | 3 | 3.13 | 0 | 0.00 | 0 | 0.00 | 3 | 3.13 | 27.00 | 20.15 | 20.36 |
| SNNP | Hadiya | Soro | 96 | 2 | 2.08 | 0 | 0.00 | 0 | 0.00 | 2 | 2.08 | 21.50 | 23.47 | 23.43 |
| SNNP | Kefa | Adiyo | 58 | 1 | 1.72 | 1 | 1.72 | 0 | 0.00 | 2 | 3.45 | 23.50 | 25.54 | 25.47 |
| SNNP | Kefa | Bita | 150 | 0 | 0.00 | 0 | 0.00 | 0 | 0.00 | 0 | 0.00 |  | 26.25 | 26.25 |
| SNNP | Kefa | Chena | 65 | 2 | 3.08 | 3 | 4.62 | 0 | 0.00 | 5 | 7.69 | 22.40 | 25.13 | 24.92 |
| SNNP | Kefa | Decha | 150 | 0 | 0.00 | 0 | 0.00 | 0 | 0.00 | 0 | 0.00 |  | 24.56 | 24.56 |
| SNNP | Kefa | Gimbo | 176 | 6 | 3.41 | 1 | 0.57 | 0 | 0.00 | 7 | 3.98 | 27.14 | 26.98 | 26.99 |
| SNNP | Kembata Timbaro | Tembaro | 48 | 2 | 4.17 | 2 | 4.17 | 0 | 0.00 | 4 | 8.33 | 20.50 | 22.34 | 22.19 |
| SNNP | Konso | Karat Zuria | 396 | 2 | 0.51 | 7 | 1.77 | 0 | 0.00 | 8 | 2.02 | 24.25 | 25.92 | 25.89 |
| SNNP | Sidama | Dara | 24 | 0 | 0.00 | 0 | 0.00 | 0 | 0.00 | 0 | 0.00 |  | 27.08 | 27.08 |
| SNNP | Sidama | Loko Abaya | 1690 | 24 | 1.42 | 77 | 4.56 | 2 | 0.12 | 96 | 5.68 | 19.09 | 23.35 | 23.10 |
| SNNP | South Omo | Bena Tsemay | 224 | 3 | 1.34 | 0 | 0.00 | 0 | 0.00 | 3 | 1.34 | 21.67 | 27.69 | 27.61 |
| SNNP | South Omo | Hamer | 73 | 0 | 0.00 | 0 | 0.00 | 0 | 0.00 | 0 | 0.00 |  | 21.38 | 21.38 |
| SNNP | South Omo | Male | 50 | 0 | 0.00 | 0 | 0.00 | 0 | 0.00 | 0 | 0.00 |  | 17.24 | 17.24 |
| SNNP | South Omo | Salamago | 246 | 0 | 0.00 | 8 | 3.25 | 0 | 0.00 | 8 | 3.25 | 19.50 | 22.16 | 22.08 |
| SNNP | West Omo | Bero | 215 | 17 | 7.91 | 4 | 1.86 | 0 | 0.00 | 20 | 9.30 | 23.15 | 27.03 | 26.67 |
| SNNP | West Omo | Maji | 275 | 13 | 4.73 | 1 | 0.36 | 0 | 0.00 | 14 | 5.09 | 21.57 | 23.90 | 23.79 |
| SNNP | West Omo | Menit Goldia | 72 | 3 | 4.17 | 0 | 0.00 | 0 | 0.00 | 3 | 4.17 | 23.00 | 26.58 | 26.43 |
| SNNP | Wolayta | Damot Woyde | 96 | 3 | 3.13 | 7 | 7.29 | 0 | 0.00 | 10 | 10.42 | 21.80 | 25.84 | 25.42 |
| SNNP | Wolayta | Duguna Fango | 200 | 5 | 2.50 | 17 | 8.50 | 0 | 0.00 | 22 | 11.00 | 17.82 | 22.60 | 22.07 |
| SNNP | Wolayta | Humbo | 1218 | 15 | 1.23 | 89 | 7.31 | 0 | 0.00 | 104 | 8.54 | 19.56 | 22.40 | 22.16 |
| SNNP | Wolayta | Kindo Didaye | 571 | 35 | 6.13 | 32 | 5.60 | 2 | 0.35 | 68 | 11.91 | 22.62 | 23.09 | 23.04 |
| SNNP | Wolayta | Kindo Koysha | 48 | 2 | 4.17 | 2 | 4.17 | 0 | 0.00 | 3 | 6.25 | 24.00 | 25.47 | 25.38 |
| SNNP | Yem Special woreda | Yem Special woreda | 222 | 3 | 1.35 | 4 | 1.80 | 1 | 0.45 | 7 | 3.15 | 24.71 | 26.27 | 26.23 |
| TOTAL |  |  | 88,003 | 1559 | 1.77 | 2685 | 3.05 | 74 | 0.08 | 4241 | 4.82 | 22.44 | 26.13 | 25.95 |
